# Mitochondrial Donation and PGT to Reduce Risk of Mitochondrial DNA Disease

**DOI:** 10.1056/NEJMoa2415539

**Published:** 2025-07-16

**Authors:** Louise A. Hyslop, Emma L. Blakely, Magomet Aushev, Jordan Marley, Yuko Takeda, Angela Pyle, Eilis Moody, Catherine Feeney, Jan Dutton, Carol Shaw, Sarah J. Smith, Kate Craig, Charlotte L. Alston, Lisa Lister, Karina Endacott, Samantha Byerley, Helen McDermott, Kathryn Wilson, Lynne Botham, Beth Matthew, Nilendran Prathalingham, Matthew Prior, Alison Murdoch, Douglass M. Turnbull, Gavin Hudson, Meenakshi Choudhary, Robert W. Taylor, Rekha N. Pillai, Jane A. Stewart, Robert McFarland, Mary Herbert

**Affiliations:** Newcastle Fertility Centre, Newcastle upon Tyne Hospitals NHS Foundation Trust; International Centre for Life, Newcastle, UK; Institute, Faculty of Medical Sciences, Newcastle University, Newcastle upon Tyne, UK; NHS Highly Specialised Service for Rare Mitochondrial Disorders, Newcastle upon Tyne Hospitals NHS Foundation Trust, Newcastle upon Tyne, UK; Biosciences Institute, Faculty of Medical Sciences, Newcastle University; Biomedicine West Wing, Centre for Life, Times Square, Newcastle upon Tyne, UK; Biosciences Institute, Faculty of Medical Sciences, Newcastle University; Biomedicine West Wing, Centre for Life, Times Square, Newcastle upon Tyne, UK; Biosciences Institute, Faculty of Medical Sciences, Newcastle University; Biomedicine West Wing, Centre for Life, Times Square, Newcastle upon Tyne, UK; Mitochondrial Research Group, Translational and Clinical Research Institute, Faculty of Medical Sciences, Newcastle University, Newcastle upon Tyne, UK; Newcastle Fertility Centre, Newcastle upon Tyne Hospitals NHS Foundation Trust; International Centre for Life, Newcastle, UK; NHS Highly Specialised Service for Rare Mitochondrial Disorders, Newcastle upon Tyne Hospitals NHS Foundation Trust, Newcastle upon Tyne, UK; Newcastle Fertility Centre, Newcastle upon Tyne Hospitals NHS Foundation Trust; International Centre for Life, Newcastle, UK; Newcastle Fertility Centre, Newcastle upon Tyne Hospitals NHS Foundation Trust; International Centre for Life, Newcastle, UK; NHS Highly Specialised Service for Rare Mitochondrial Disorders, Newcastle upon Tyne Hospitals NHS Foundation Trust, Newcastle upon Tyne, UK; NHS Highly Specialised Service for Rare Mitochondrial Disorders, Newcastle upon Tyne Hospitals NHS Foundation Trust, Newcastle upon Tyne, UK; Mitochondrial Research Group, Translational and Clinical Research Institute, Faculty of Medical Sciences, Newcastle University, Newcastle upon Tyne, UK; NHS Highly Specialised Service for Rare Mitochondrial Disorders, Newcastle upon Tyne Hospitals NHS Foundation Trust, Newcastle upon Tyne, UK; Newcastle Fertility Centre, Newcastle upon Tyne Hospitals NHS Foundation Trust; International Centre for Life, Newcastle, UK; Newcastle Fertility Centre, Newcastle upon Tyne Hospitals NHS Foundation Trust; International Centre for Life, Newcastle, UK; Newcastle Fertility Centre, Newcastle upon Tyne Hospitals NHS Foundation Trust; International Centre for Life, Newcastle, UK; Newcastle Fertility Centre, Newcastle upon Tyne Hospitals NHS Foundation Trust; International Centre for Life, Newcastle, UK; Biosciences Institute, Faculty of Medical Sciences, Newcastle University; Biomedicine West Wing, Centre for Life, Times Square, Newcastle upon Tyne, UK; Newcastle Fertility Centre, Newcastle upon Tyne Hospitals NHS Foundation Trust; International Centre for Life, Newcastle, UK; Newcastle Fertility Centre, Newcastle upon Tyne Hospitals NHS Foundation Trust; International Centre for Life, Newcastle, UK; Newcastle Fertility Centre, Newcastle upon Tyne Hospitals NHS Foundation Trust; International Centre for Life, Newcastle, UK; Newcastle Fertility Centre, Newcastle upon Tyne Hospitals NHS Foundation Trust; International Centre for Life, Newcastle, UK; Newcastle Fertility Centre, Newcastle upon Tyne Hospitals NHS Foundation Trust; International Centre for Life, Newcastle, UK; Newcastle Fertility Centre, Newcastle upon Tyne Hospitals NHS Foundation Trust; International Centre for Life, Newcastle, UK; Mitochondrial Research Group, Translational and Clinical Research Institute, Faculty of Medical Sciences, Newcastle University, Newcastle upon Tyne, UK; Mitochondrial Research Group, Biosciences Institute, Faculty of Medical Sciences, Newcastle University, Newcastle upon Tyne, UK; Newcastle Fertility Centre, Newcastle upon Tyne Hospitals NHS Foundation Trust; International Centre for Life, Newcastle, UK; Mitochondrial Research Group, Translational and Clinical Research Institute, Faculty of Medical Sciences, Newcastle University, Newcastle upon Tyne, UK; NHS Highly Specialised Service for Rare Mitochondrial Disorders, Newcastle upon Tyne Hospitals NHS Foundation Trust, Newcastle upon Tyne, UK; Newcastle Fertility Centre, Newcastle upon Tyne Hospitals NHS Foundation Trust; International Centre for Life, Newcastle, UK; Newcastle Fertility Centre, Newcastle upon Tyne Hospitals NHS Foundation Trust; International Centre for Life, Newcastle, UK; Mitochondrial Research Group, Biosciences Institute, Faculty of Medical Sciences, Newcastle University, Newcastle upon Tyne, UK; Mitochondrial Research Group, Translational and Clinical Research Institute, Faculty of Medical Sciences, Newcastle University, Newcastle upon Tyne, UK; NHS Highly Specialised Service for Rare Mitochondrial Disorders, Newcastle upon Tyne Hospitals NHS Foundation Trust, Newcastle upon Tyne, UK; Newcastle Fertility Centre, Newcastle upon Tyne Hospitals NHS Foundation Trust; International Centre for Life, Newcastle, UK; Department of Anatomy and Developmental Biology, Monash Biomedicine Discovery Institute, Monash University; Melbourne, Australia

## Abstract

**Background:**

Children born to women who carry pathogenic variants in mitochondrial DNA (mtDNA) are at risk of developing a range of clinical syndromes collectively known as mtDNA disease. Mitochondrial donation by pronuclear transfer involves nuclear genome transplantation from a fertilized carrier egg to an enucleated fertilized egg donated by an unaffected woman. Thus, pronuclear transfer offers affected women the potential to have a genetically related child with a reduced risk of developing mtDNA disease.

**Methods:**

We offered mitochondrial donation (through pronuclear transfer) or preimplantation genetic testing (PGT) to a series of affected women who sought to reduce transmission of pathogenic mtDNA variants to their children. Patients with heteroplasmy were offered PGT and patients with homoplasmy or elevated heteroplasmy were offered pronuclear transfer.

**Results:**

Clinical pregnancies were confirmed in 8 of 22 (36%) and 16 of 39 (41%) of patients who underwent an intracytoplasmic sperm injection procedure for pronuclear transfer or PGT, respectively. Pronuclear transfer resulted in 8 live births and 1 ongoing pregnancy. PGT resulted in 18 live births. Heteroplasmy in the blood of the 8 infants resulting from pronuclear transfer ranged from undetectable to 16%, a reduction of approximately 77.6-100% compared with the corresponding enucleated zygotes. Infant heteroplasmy after PGT, known for 10 of the 18 infants, ranged from undetectable to 7%.

**Conclusion:**

Mitochondrial donation through pronuclear transfer is compatible with human embryo viability. An integrated programme of pronuclear transfer and PGT is effective in reducing transmission of homoplasmic and heteroplasmic pathogenic mtDNA variants.

(Funded by NHS England and others.)

## Introduction

The mitochondrial genome is maternally inherited and children born to women who carry pathogenic mitochondrial DNA (mtDNA) variants are at risk of developing life-limiting mtDNA disease^1,2^. MtDNA disorders have an estimated birth prevalence of 1 in 5000^2^. Pathogenic variants can be either homoplasmic (present in all copies of mtDNA) or heteroplasmic (present in a proportion of mtDNAs). Homoplasmic variants are transmitted in full to all children, but the penetrance of homoplasmic variants varies^3^. Transmission of heteroplasmic variants is subject to a genetic bottleneck, resulting in widely varying heteroplasmy between eggs from the same woman^3^, making it difficult to predict the risk of a child developing severe disease^4^.

In the absence of curative treatments, advances in assisted reproductive technology (ART) offer the possibility to reduce transmission of pathogenic mtDNA variants^5–7^. PGT, which is widely used for detecting nuclear genetic defects^6,8^, can be used to reduce the risk of mtDNA disease by identifying embryos with low levels of the maternal mtDNA variant^9^. In the event that there is no embryo with low heteroplasmy available, mitochondrial donation (or mitochondrial replacement) offers an alternative means of potentially reducing the risk of mtDNA disease: this process involves the microsurgical transplantation of the nuclear genome from a carrier egg to an unaffected enucleated egg^5,7,10^.

Mitochondrial donation can be performed before fertilization (during metaphase II arrest) or after fertilization when the maternal and paternal haploid genomes are contained in pronuclei^10^. Both approaches (maternal-spindle transfer and pronuclear transfer) can result in embryos with heteroplasmy for maternal mtDNA owing to co-transfer (carryover) of some mitochondria surrounding the transplanted pronuclei. ^11–13^ For reasons that remain unclear, the small fraction of maternal mtDNA increases to become homoplasmic in ~20% of embryonic stem cell lines derived from mitochondrial-donation embryos. ^11,14,15^ Moreover, elevated heteroplasmy (40-60%) for maternal mtDNA has been reported in 1 of 6 babies born after maternal-spindle transfer for infertility treatment^16^. These findings raised questions about whether mitochondrial donation can reliably prevent transmission of pathogenic mtDNA variants in all cases, particularly in cases of homoplasmy^17^. Here we demonstrate the clinical application of a pronuclear-transfer procedure previously optimised for human zygotes^11^. Pronuclear transfer and PGT were offered in an integrated program to reduce transmission of a range of heteroplasmic and homoplasmic pathogenetic variants. Maternal and child health outcomes are described by McFarland et al. in an accompanying report in this issue of the *Journal*.^ref^

## Methods

### Materials and Methods

ART treatment procedures including pronuclear transfer were conducted under Human Fertilisation and Embryology Authority (HFEA) Centre License 0017. Clinical pronuclear-transfer procedures were performed by an HFEA-licensed pronuclear-transfer practitioner. Patient-specific approval for pronuclear-transfer treatment was obtained from the HFEA Statutory Approvals Committee. Research was approved by the Newcastle and North Tyneside Research Ethics Committee and was licensed by the HFEA (R0152).

PGT and pronuclear-transfer patients underwent health assessments and standard fertility assessments prior to undergoing ovarian stimulation and oocyte retrieval. In addition, egg donor mtDNA was sequenced to screen for pathogenic mtDNA variants. All patients and donors were offered counselling before entering the program and throughout the treatment pathway (Supplementary Methods).

**Figure 1:**
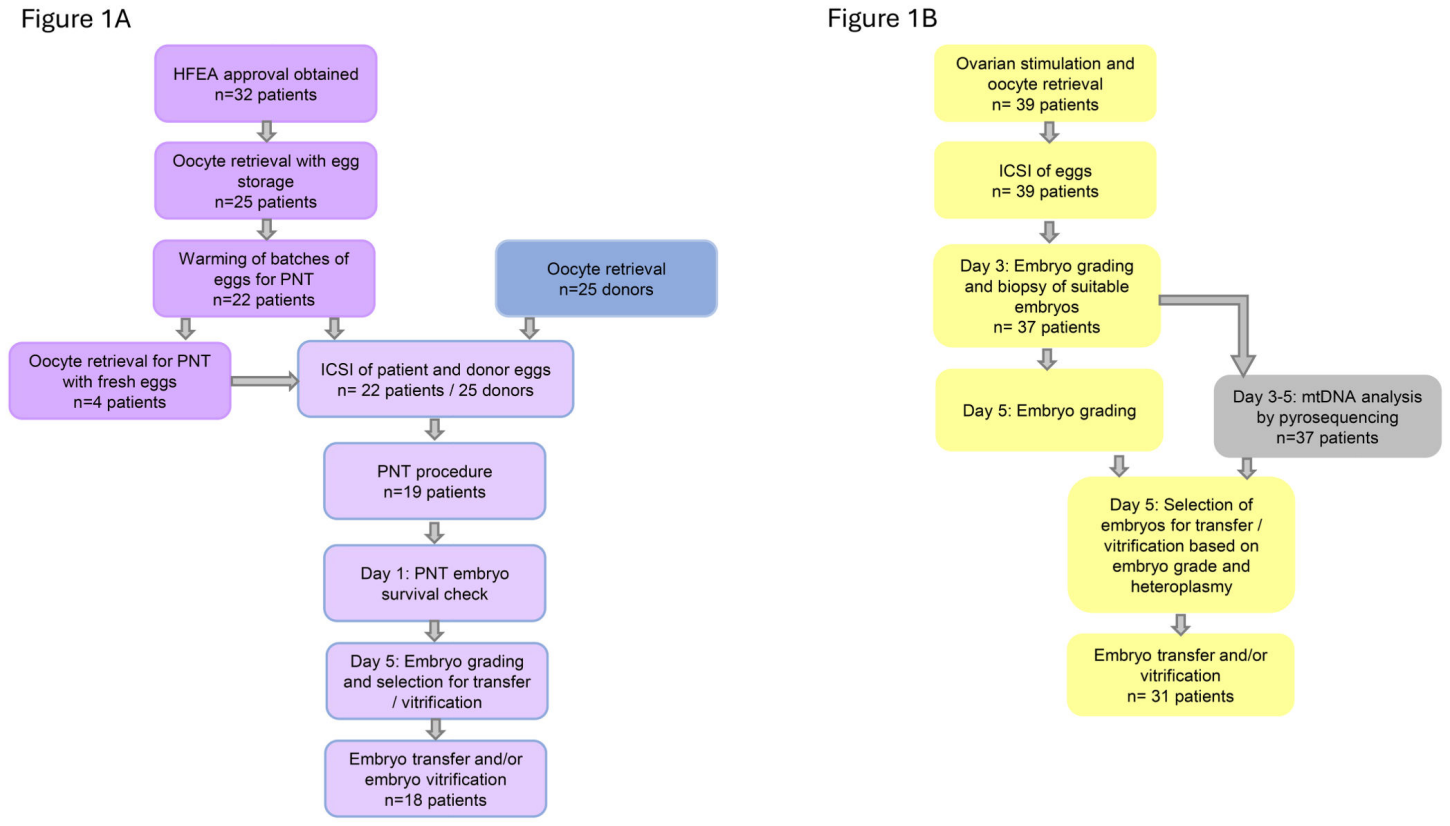
pronuclear transfer and PGT treatment pathways. **(A)** Schematic of the pronuclear-transfer pathway and progression of the 32 patients approved through the stages of egg storage, pronuclear transfer treatment and embryo transfer. **(B)** Schematic of the PGT pathway and progression of the 39 patients through the stages of ICSI, embryo biopsy and embryo transfer.

Based on evidence from preclinical research^11^, the standard practice is to vitrify pronuclear-transfer patient eggs and store them until fresh donor eggs become available. All eggs were fertilized by intracytoplasmic sperm injection (ICSI). Pronuclear transfer was performed at 8-13 hours after ICSI and intact zygotes were cultured for 5-6 days. For PGT treatment, a single blastomere was removed on day 3 for quantitative analysis of mtDNA heteroplasmy^18^ and embryos were cultured for a further 2-3 days. Embryos of suitable quality were used for fresh intrauterine transfer or vitrified for a future frozen embryo transfer. In the case of PGT, those with <30% heteroplasmy were preferentially used for treatment (Fig 1A and B). An assessment of heteroplasmy for the maternal pathogenic variant was determined in infant peripheral blood (Supplementary methods).

Data were analyzed using Chi-square test, Fisher’s exact test, unpaired t test and Tukey’s multiple comparison test in GraphPad Prism v9.0.1, as indicated in the figure legends. Means presented are ± the standard deviation (sd).

## Results

### Patients

Thirty-two patients have been granted case-specific HFEA approval for pronuclear-transfer treatment. We describe here the treatment outcomes of women who have undergone an oocyte retrieval procedure for pronuclear-transfer treatment (n=25) or who underwent PGT to reduce transmission of pathogenic mtDNA variants (n=39) (Table S1; Fig. 1). The patients were predominantly of White European ethnicity (Table S2).

Mitochondrial DNA encodes 13 respiratory chain complex subunits, 22 tRNAs (mt-tRNA) and 2 rRNAs^1^. Among patients who have commenced treatment, pathogenic variants were present in either protein coding (n=27) or mt-tRNA (n=33) genes and were homoplasmic (n=12) or heteroplasmic (n=48). One patient had a large-scale mtDNA deletion (NC_012920.1: m.7529_14025del). PGT was offered in most cases of heteroplasmy (n=39) and pronuclear transfer was offered in cases of homoplasmy (n= 12) and in some cases of high heteroplasmy (n=13; Fig. S1). The latter included 3 patients who were offered pronuclear transfer having previously undergone at least one cycle of PGT as part of this study.

Pathogenic variants in protein-coding mtDNA sequences were present in *MT-ND* genes (encoding complex I subunits; n=17 patients) or in *MT-ATP6* (encoding a complex V subunit; n= 9 patients). *MT-ND* gene variants included Leber Hereditary Optic Neuropathy (LHON) variants (n=11) and non-LHON variants (n=6). LHON variants are typically homoplasmic and LHON carriers accounted for the largest cohort (44%) of pronuclear-transfer patients. All other protein-coding variants were heteroplasmic; women carrying these variants underwent PGT. Similarly, most patients carrying mt-tRNA variants (69.7%) underwent PGT treatment.

The m.3243A>G *MT-TL1* variant was most common, and 15 of the 21 women who carried this variant underwent PGT treatment. Pronuclear transfer was offered in cases of elevated heteroplasmy for the m.3243A>G *MT-TL1* variant (n= 5) and for the m.8344A>G *MT-TK* variant (n=4; Fig. S1), including 3 patients who had previously undergone PGT treatment as part of this study. Five pronuclear-transfer patients had homoplasmy (n=4) or elevated heteroplasmy (n= 1) for mt-tRNA variants referred to here as “rare mt-tRNA variants” (Fig. S1).

### Oocyte retrieval and fertilization

**Figure 2:**
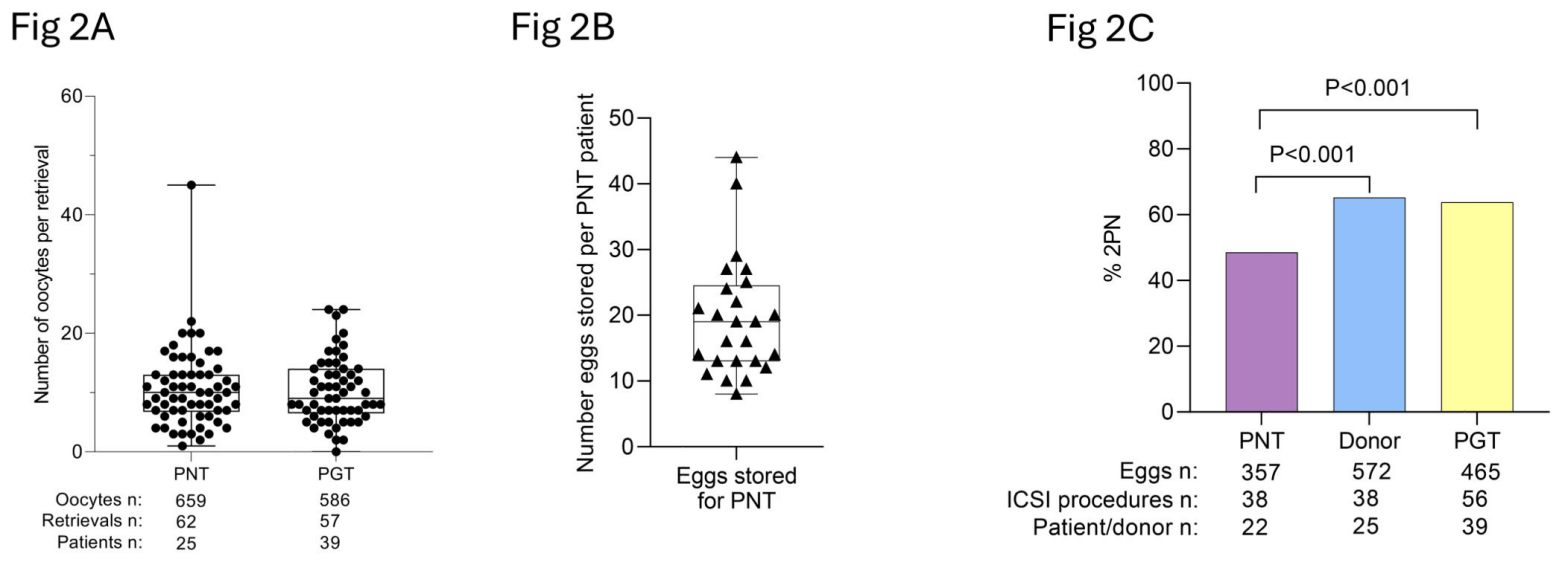
Oocyte retrieval and fertilization outcomes of pronuclear transfer patient, PGT patient and donor eggs. **(A)** Number of oocytes per egg retrieval for pronuclear-transfer and PGT patients. **(D)** Number of oocytes per egg retrieval for egg donors. **(B) (C)** Comparison of proportions of normally fertilized patient (pronuclear transfer and PGT) and donor eggs (P<0.001, Chi-Square).

The age of the participants was similar between the PGT (median 34 years; range 22-40 years) and pronuclear transfer (median 34 years; range 25-40 years) groups (Fig. S2A). Both patient cohorts underwent 1-3 ovarian-stimulation and oocyte-retrieval procedures (Fig. S3A and B) resulting in 659 (mean 10.6 ± 6.7) and 586 (mean 10.3 ± 5.5) oocytes respectively (Fig. 2A). The number of oocytes retrieved was similar between PGT, pronuclear transfer and age-matched control patients (Fig. S2B and C). Twenty-five egg donors (aged 21-37 years) underwent ovarian stimulation and egg retrieval (n=38 procedures), resulting in 736 (mean 19.4 ± 8.7) oocytes (Fig. S3C). The proportion of mature oocytes (eggs) at the time of retrieval was equivalent between the two patient cohorts (79.4% and 78.8% for PGT and pronuclear transfer respectively) and the egg donors (80.2%) (P=0.72 and P=0.52 respectively, Chi-Square; Fig. S3D).

Eggs (n=487) obtained from the pronuclear-transfer patients (n=25) were vitrified and placed in cryostorage (Fig. 2B). Of these 487 vitrified eggs, 327 (from 22 patients) were removed from storage in batches of 3-14 eggs on the day of the donor oocyte retrieval (Fig. S3E); 325 (99.4%) survived the vitrification/warming procedure (Fig. S3F). Four women had fresh (n=32) and vitrified (n=16) eggs used for pronuclear transfer (Fig. 1A). Fresh eggs were used because of concerns about fertilization (n=1) and endometrial thickness (n=1) in a previous cycle, or to expedite treatment (n=2).

ICSI was performed on the pronuclear-transfer patients’ eggs (n= 357) using patient-partner (n= 21) or donor (n=1) sperm. Donor eggs (n=572) and corresponding participant eggs were injected with sperm from the same source. However, in 3 cases, the use of donor sperm (ie, from a different source) to fertilize donor eggs was mandated because the egg donor was genetically related to the male partner.

Normal fertilization, evidenced by the presence of 2 pronuclei, was observed in 48.5% of pronuclear-transfer patient eggs compared with 63.8% of donor eggs (P<0.001; Chi-Square; Fig. 2C) and 66.5% of PGT patient eggs (P<0.001; Chi-Square; Fig. 2C). Fertilization of pronuclear-transfer patient eggs may have been affected by vitrification (Fig. S4A and B). However, we observed variation in success in fertilization according the pathogenic variant (Fig. S3C and D). Three patients had no fertilized eggs available for pronuclear transfer (Table S3).

### Pronuclear transfer, embryo biopsy and development

**Figure 3:**
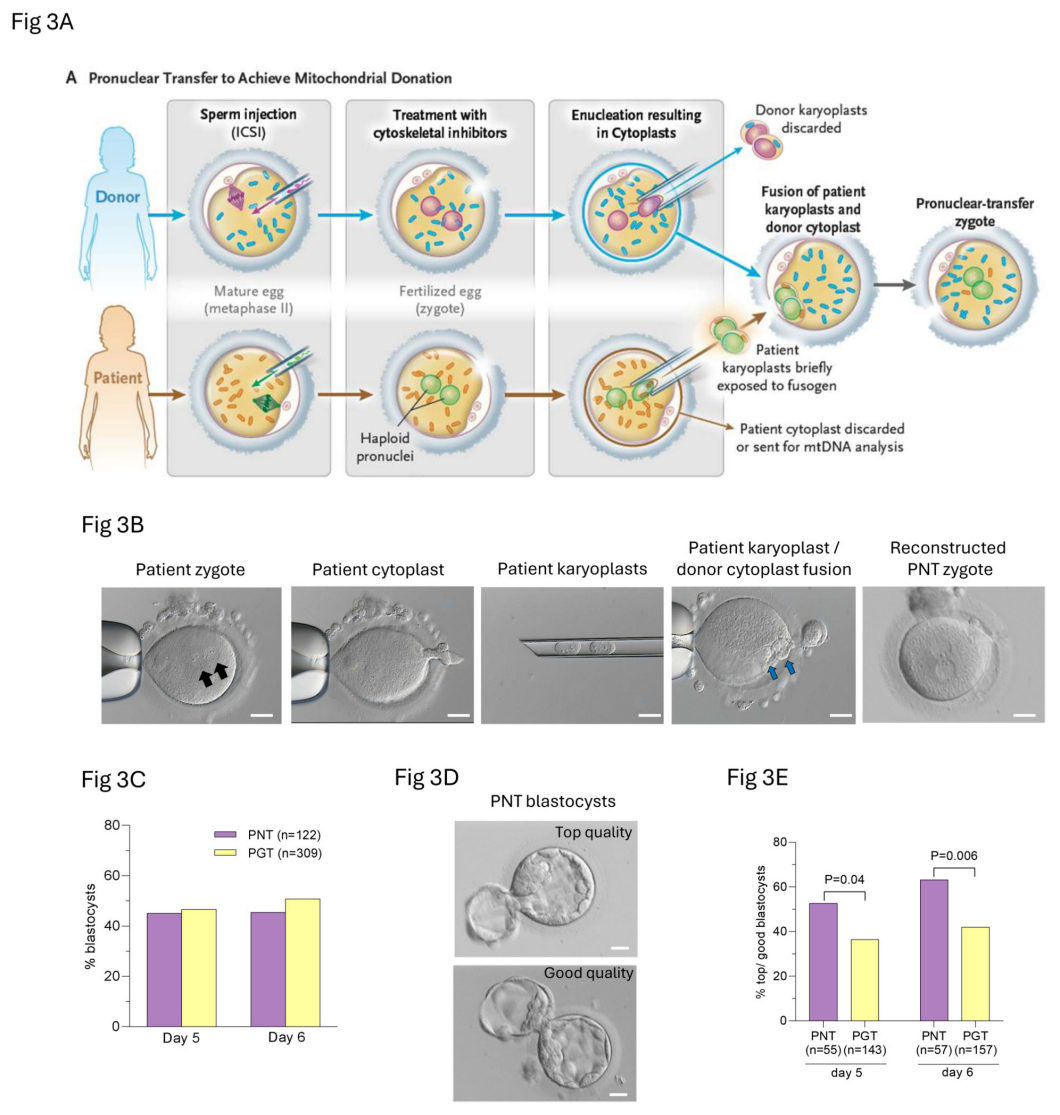
pronuclear transfer procedure and comparison of pronuclear transfer and PGT embryo development. **(A)** Schematic illustrating the pronuclear-transfer procedure. **(B)** Images showing enucleation and fusion of patient karyoplast and donor egg cytoplast; scale bar = 20µm. **(C)** Comparison of proportions of pronuclear-transfer and PGT embryos developing to the blastocyst stage. **(D)** Images showing examples of top and good quality pronuclear-transfer blastocysts; scale bar = 20µm. **(E)** Comparison of the proportions of top and good quality blastocysts after pronuclear transfer and PGT showing an increased proportion of top and good quality pronuclear-transfer blastocysts on day 5 (P=0.04; Chi-square) and day 6 (P=0.006; Chi-square).

Pronuclear transfer was performed at 8-13 hours after ICSI^11^ between 160 pairs of normally fertilized zygotes from 19 pronuclear-transfer patients and 24 egg donors. Pronuclei were removed separately and placed together under the zona of the enucleated donor egg after brief exposure to a fusion agent (hemagglutinating Virus of Japan envelope) (Fig. 3A and B; Movie 1). To provide an indicator of mitochondrial donation–induced heteroplasmy, semiquantitative scores were assigned to estimate the volume of cytoplasm contained in patient karyoplasts (Fig. S5A and C). A score was also assigned for the volume of cytoplasmic leakage from donor eggs (Fig. S5B and C). Replacement of the donor zygote pronuclei with the pronuclei from the patient’s zygote was successful in 127 of 160 (79.4%) attempts, 122 of the 127 (96.1%) embryos resulting from these successful attempts were intact on the next day (day 1; Fig. S6A). The number of intact zygotes per pronuclear transfer procedure (n=33) ranged from 0-7 (Fig. S6B; Table S3).

PGT embryos were assessed on day 3 after ICSI and those with ≥6 cells (n=262; 84.8% of zygotes) from 37 (94.9%) patients had a single blastomere removed^18^ for mtDNA analysis. Blastocyst formation was comparable between pronuclear transfer and PGT zygotes on days 5 (45.1% *vs* 46.3%; P=0.82, Chi-Square) and 6 (46.7% *vs* 50.8%; P=0.44, Chi-Square, Fig 3C). The proportion of ‘top’ and ‘good’ quality blastocysts^19^ was reduced for PGT compared with pronuclear transfer (42% *vs* 63.2% on day 6, P=0.006; Chi-Square; Fig. 3D and E).

Our current practice is to preferentially exclude blastocysts with >30% heteroplasmy from use in treatment. However, decision-making varies on selection or exclusion of blastocysts depending on the variant and family history. In this series, 42.4% of ‘top’ and ‘good’ quality PGT blastocysts had >30% heteroplasmy (Fig. S6C) and were therefore not used for treatment. Thus, the best-quality PGT embryos could not be used for treatment in 25% of fresh embryo transfers.

### Embryo transfer and treatment outcomes

**Figure 4:**
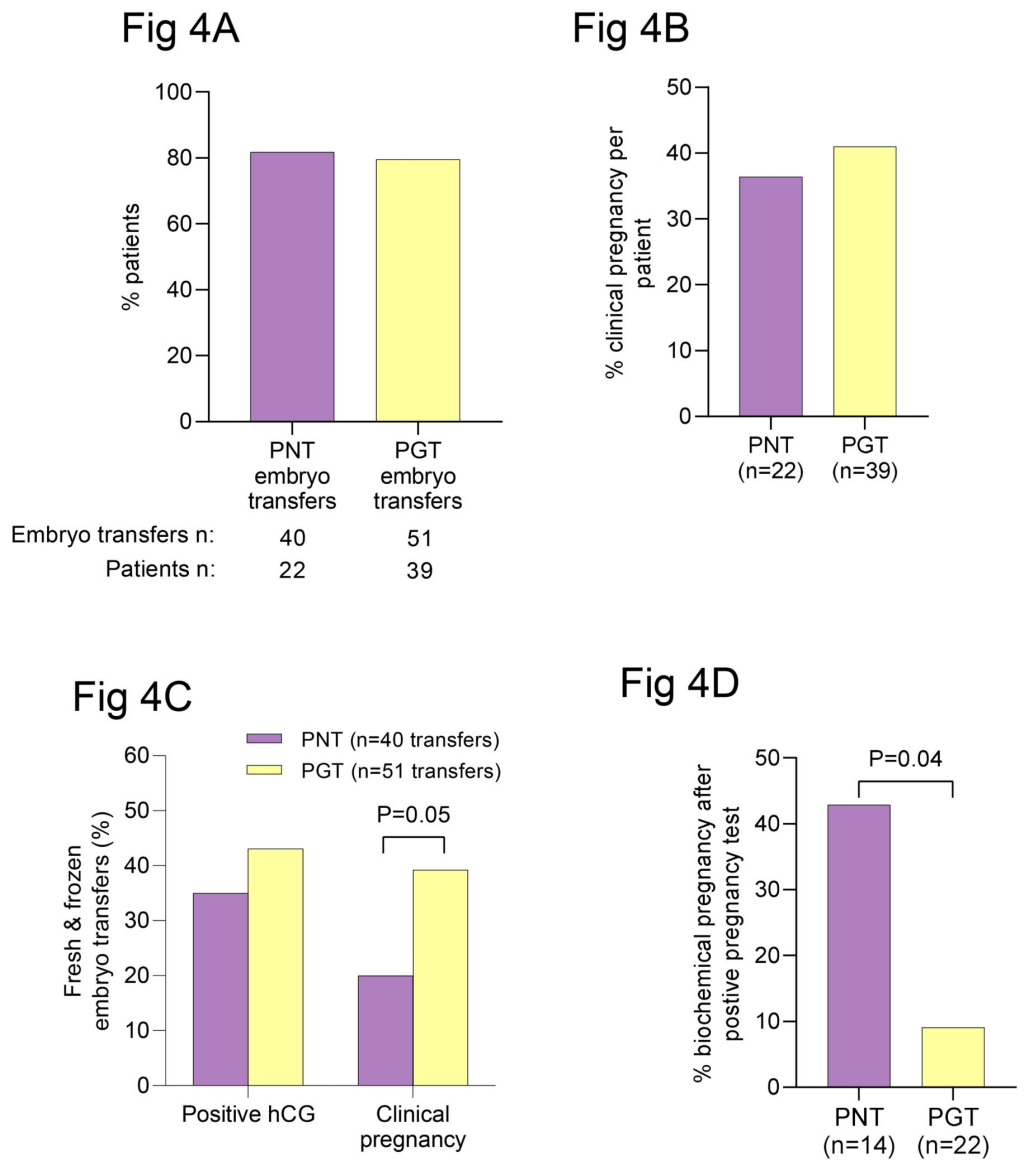
Clinical outcomes after pronuclear transfer and PGT. **(A)** Proportion of patients who had embryos available for intrauterine transfer after ICSI for pronuclear transfer or PGT. **(B)** Graph showing the overall proportion of patients who has a clinical pregnancy after pronuclear transfer and PGT. **(C)** Graph showing the proportions embryo transfers (fresh and frozen) that resulted in a positive pregnancy test and clinical pregnancy after pronuclear transfer and PGT. Pronuclear-transfer patients had a reduced incidence of clinical pregnancy (P=0.05; Chi-Square). **(D)** Graph showing an increased proportion of biochemical pregnancies after pronuclear transfer (P=0.04; Fisher’s exact).

The proportion of ICSI procedures resulting in a fresh or frozen embryo transfer procedure was 73.7% for pronuclear transfer and 66.7% for PGT (Fig. S7A). In 5 cases, all suitable embryos were vitrified owing to concerns regarding the risk of ovarian hyperstimulation or poor endometrial response after hormonal priming for embryo transfer. Of the patients who underwent an ICSI procedure for pronuclear transfer (n= 22) or PGT (n=39), 82% and 80% respectively had embryos available for a fresh and/or frozen embryo transfer procedure (Fig. 4A). The predominant reasons for having no embryos available for transfer were failure of fertilization for pronuclear transfer patients (n= 3) and failure to obtain low-load embryos for replacement for PGT patients (n= 5; Fig. S7B). Pronuclear-transfer patients had a single embryo replaced in all embryo transfer procedures (n=40). A single embryo was replaced in 42 of 51 PGT patient embryo transfer procedures; 2 embryo were replaced in 9 procedures owing to concerns about female age (n = 1) or embryo quality (n= 8)(Fig. S7C).

The overall incidence of clinical pregnancy (confirmed by ultrasound scan at 7 weeks of gestation) per patient who had an ICSI procedure was similar in the two groups (36.4%; n=22 pronuclear-transfer patients and 41%; n=39 PNT patients; P=0.72, Chi-Square; Fig. 4B). However, the incidence of clinical pregnancy per embryo transfer (fresh and frozen Fig. S7D and E) was reduced for pronuclear transfer compared with PGT (20% vs 39.2%; P=0.05; Chi-Square; Fig 4C). Consistent with this, the incidence of pregnancy loss after a positive pregnancy test was greater for pronuclear-transfer cases compared with PGT cases (P=0.04; Fisher’s exact; Fig. 4D), particularly after transfer of fresh pronuclear transfer embryos (P=0.02; Fisher’s exact; Fig. S7F). Three of six patients who had a biochemical pregnancy had a viable clinical pregnancy after a subsequent embryo transfer; in two of these three patients, the earlier biochemical pregnancy and the later clinical pregnancy involved eggs from the same donor. Thus, the reduced viability of pronuclear-transfer embryos is not readily explained by intrinsic maternal factors or by an effect of potentially unfavorable nuclear/mitochondrial combinations. Three of the confirmed PGT clinical pregnancies (n=20) ended in miscarriage (15%). None of the pronuclear transfer pregnancies (n=8) ended in miscarriage (Table S4).

Five of the 8 patients who had a clinical pregnancy after pronuclear transfer carried primary LHON variants and 3 carried rare mt-tRNA variants (Fig. S7G; Table S3). None of the pronuclear-transfer patients carrying the m.3243A>G (n= 3) and m.8344A>G (n=4) variants had a clinical pregnancy. Of the 16 patients who had a clinical pregnancy after PGT, half carried protein coding variants (*MT-ATP6* gene variants (n=4); non-LHON *MT-ND* gene variants (n=4)) and half carried mt-tRNA variants (m.3243A>G (n=6) and m.8344A>G (n=2); Fig. S7H; Table S3). These findings demonstrate the success of both approaches in achieving viable pregnancies in women with a wide range of mtDNA variants.

### Live Births

Six of the 21 patients who had a live birth after PGT or pronuclear-transfer treatment have previously given birth to an affected child (Table S3). In this study, pronuclear transfer has resulted in 8 live births (4 female and 4 male infants, including monozygotic twins) and 1 ongoing pregnancy. PGT has resulted in 18 live births (9 female and 9 male infants; Fig. S8A). One PGT patient gave birth to 1 set of dizygotic twins and 3 PGT patients had 2 singleton live births (Table S3). There was no significant difference in the gestational age between pronuclear transfer and PGT (P=0.41, unpaired t test; Fig. S8B). Similarly, analysis of birth weights according to gestational age showed no difference between the two treatments (Fig. S8C; Fisher’s Exact). There were no reports of congenital abnormalities at birth. However, a cardiac anomaly was detected in one infant born after PGT for the m.3243A>G variant and in one born after pronuclear transfer for the m.4300G>A variant. In both cases, infant heteroplasmy for the maternal pathogenic variant was ≤5%.

### Heteroplasmy After Pronuclear Transfer and PGT

Regulations in the UK do not permit assay of heteroplasmy in embryos obtained by pronuclear transfer (HFEA Code of Practice Section 33, Edition 9.4. October 2023). Moreover, none of the pronuclear-transfer patients included in this series opted for prenatal diagnosis. In 5 of 8 newborns heteroplasmy for the maternal pathogenic variant in blood spot tests (Guthrie card) was undetectable (<3%) by quantitative pyrosequencing. High-throughput DNA sequencing of DNA isolated from blood spots of 3 of these 5 showed the presence of heteroplasmy ranging from 0.06-0.17% (Table 1). Three newborns had heteroplasmy of 5%, 12% and 16% (Table 1).

High-throughput DNA sequence analysis of pronuclear-transfer embryos not suitable for use in treatment (n=62) revealed consistently low heteroplasmy (ranging from undetectable to 3.9%; mean 1.18 ± 0.90 s.d.; Fig. S9A). regardless of whether the corresponding enucleated patient zygote was homoplasmic or heteroplasmic (Fig. S9B).

Analysis of variant heteroplasmy in arrested pronuclear-transfer embryos according to the scores assigned for carryover of maternal cytoplasm during pronuclear transfer (where 1 is low and 4 is high), showed a correlation between carryover scores and embryo heteroplasmy (P<0.01; Tukey’s multiple comparison test; Fig. S9C). Carryover scores of 1 and 2 were assigned for the pronuclear-transfer procedures that resulted in 12% and 16% neonatal heteroplasmy respectively (Table 1). This is comparable with scores assigned for pronuclear-transfer procedures that resulted in <1% heteroplasmy (detected by NGS) in infant blood (Table 1). Moreover, heteroplasmy in embryos with carryover scores of 1 (n=24 embryos) and 2 (n=24 embryos) ranged from 0 to 1.6% (mean 0.74 ± 0.48 s.d) and from 0 to 2.8% (mean 0.99 ± 0.69 s.d) respectively (Fig. S9C). Thus, it is unlikely that increased carryover of maternal cytoplasm accounts for the relatively high neonatal heteroplasmy (12% and 16%) observed in 2 pronuclear-transfer cases.

PGT patients are encouraged to have child heteroplasmy measured but not all pursue this option. In the current series, heteroplasmy data were available for 10 of 18 children born after PGT treatment. In 8 cases involving single embryo transfer, the pathogenic variant was undetectable in the test blastomere and in infant blood (Table 1). Two children were singletons born after the transfer of 2 embryos, and it was not possible to establish which embryo survived to birth. In one of these cases, infant heteroplasmy was below the limit of detection following transfer of 2 embryos with 9% and 24% heteroplasmy in the test blastomeres (Table 1). In the other, infant heteroplasmy was 7%.

The decision of whether to offer pronuclear transfer or PGT was based on the probability of obtaining embryos with <30% heteroplasmy. Eleven patients with heteroplasmy (67% to 98%) elected to undergo at least one pronuclear-transfer procedure. Analysis of enucleated zygotes (n=67) from 10 of these patients showed that the majority (70%) had no zygotes with <30% heteroplasmy. Three of the women each had a single zygote with <30% heteroplasmy (range 12-25%; Fig. S10A). Overall, 4.5% of enucleated zygotes had <30% heteroplasmy and 67.2% with >60% (Fig. S10B), which is associated with an increased risk of developing disease symptoms^9^. Thus, while it is difficult to rule out the possibility of pronuclear-transfer patients obtaining zygotes with low heteroplasmy, it is unlikely that those who underwent pronuclear transfer would have benefited from PGT.

## Discussion

Building on our previous preclinical findings^11^, we demonstrate that clinical translation of pronuclear transfer is compatible with human embryo viability and reduces transmission of pathogenic mtDNA variants; analyses of neonatal blood support reduced levels of maternal pathogenic mtDNA variant by 95-100% in 6 newborns and by 76.5-88% in 2 others. These data indicate that pronuclear transfer is effective in reducing transmission of mtDNA disease.

Considering the potential for the amplification of the small fraction of maternal mtDNA co-transferred with the nuclear genome,^11,14–16^ it will be essential to monitor outcomes in a larger series to determine whether heteroplasmy remains stable over time and across different tissue types. For two babies born after pronuclear transfer, we detected 12% and 16% heteroplasmy for the maternal pathogenic variant. Although considered to be below the critical threshold for disease symptoms^9^, it will be important to understand the technical and/or biological basis for the higher levels of variant heteroplasmy compared with the other cases reported here. Comparison of patient karyoplast scores indicated no marked difference in the amount cytoplasm surrounding the pronuclei. Potential explanations include a replicative advantage of patient mtDNA over the egg donor mtDNA^15,17^ and/or unequal distribution of mitochondrial donation-induced heteroplasmy,^11,20–22^ resulting in enrichment in a subset of embryonic cells that subsequently segregate to the epiblast lineage^23^.

Mitochondrial donation involves creating new combinations of mitochondrial and nuclear genomes. Studies involving substitution of mouse mtDNA by backcrossing the nuclear genome of one inbred strain onto the cytoplasm of another^24–27^ indicate a range of adverse effects, including on physical performance^28^ and cognition^26^, whereas others have reported beneficial effects on metabolism and longevity^27^. However, the clinical relevance is unclear as human reproduction frequently involves combining nuclear and mitochondrial genomes from diverse ancestries with no evidence of co-transmission of mtDNA and nuclear-encoded mitochondrial genes^29,30^. Moreover, population studies indicate that the co-existence of nuclear and mitochondrial genomes from diverse ancestries does not negatively impact human health^31,32^.

In accordance with the principle of cautious clinical application^7^, pronuclear transfer is offered only in cases for which PGT is unlikely to be successful. The series of 39 PGT cases resulting in 18 live births with heteroplasmy ranging from undetectable to 7% (based on 10 babies) adds to the existing body of evidence^33–38^ and confirm the reliability of PGT in reducing the risk of mtDNA-related disease.

PGT outcomes may be further improved by performing biopsy of trophectoderm cells at the blastocyst stage rather than on day 3^39^. However, reports of a clinically relevant discrepancy in heteroplasmy^40^ between biopsied trophectoderm cells and the corresponding infant^41^ highlights the importance of further investigation to test the reliability of trophectoderm biopsy across a range of heteroplasmy levels^5,6^.

Although the incidence of clinical pregnancy per patient was similar between the two groups, pronuclear transfer was less efficient than PGT in establishing clinical pregnancies owing to an increased incidence of embryo loss after a positive pregnancy test. The success of pronuclear-transfer treatment was also curtailed by a reduced proportion of normally fertilized eggs. Vitrification of patients’ eggs (which was carried out for most pronuclear-transfer procedures) may contribute to lower fertilization, however the effect varied between mtDNA variant types. This raises the possibility that the ability of an egg to undergo normal fertilization is compromised by the extent of biochemical defect caused by the variant it carries. It would be interesting to determine whether outcomes can be improved by performing spindle transfer, which would enable fertilization to occur in a wildtype cytoplasmic environment.

In conclusion, our results show that a program of PGT and pronuclear transfer is effective in reducing transmission of a range of pathogenic mtDNA variants. The reduced heteroplasmy in infants born to women carrying homoplasmic variants provides grounds for optimism. However, until more is known about its efficacy, mitochondrial donation should be regarded as a risk reduction strategy. In addition, clinical follow-up of children born after mitochondrial donation will be essential for monitoring the safety and efficacy of this procedure.

## Supplementary Material

Supplement

Supplementary Video 2

Supplemetary Video 1

Supplementary Video Legends

## Figures and Tables

**Table 1:** Infant heteroplasmy for maternal pathogenic variants. Table shows in purple the heteroplasmy levels in maternal urine, patient enucleated egg and peripheral blood (blood spot test) of infants (n=8) born to date after PNT. Table shows in yellow the heteroplasmy levels detected in maternal urine, the test blastomere and peripheral blood of children (n=10) born after PGT. mtDNA analysis was not available for 7 children born after PGT. mtDNA variant positions are in reference to the revised Cambridge reference sequence (NC_012920.1).

PNT								
Variant	Reproductive history	% heteroplasmy		PNT score		% heteroplasmy in neonatal blood		Sex
		Maternal urine	Enucleated patient egg			Pyrosequencing	NGS	
m.4300A>G	No pregnancies or births	Homoplasmic	-	2	2	5%	-	Female
m.4300A>G	Live birth of affected child (deceased)	Homoplasmic	-	2	2	Not detected*	0.06%	Male
m.3260A>G	No pregnancies or births	79%	68%	2	1	16%	-	Male
m.3460G>A	No pregnancies or births	89%	99%	2	4	Not detected*	0.17%	Female
m.11778G>A	No pregnancies or births	82%	64%	1	0	Not detected*	0.09%	Female
m.11778G>A	No pregnancies or births	Homoplasmic	-	3	1	Twin 1: Not detected*	-	Identical twin males
						Twin 2: Not detected*	-	
m.11778G>A	Live birth	98%	99.9%	1	3	12%	-	Female
**PGT**								
**Variant**		**% heteroplasmy**				**% heteroplasmy**		**Sex**
		**Maternal urine**	**Test blastomere**			**Neonatal blood** **(pyrosequencing)**		
m.10158T>C	Live birth of affected child (deceased)	33%	Not detected*			Not detected*		Male
			Not detected*			Not detected*		Female
m.13094T>C	Live birth of affected child (deceased)	45%	Not detected*			Not detected*		Male
m.3243A>G	No pregnancies or births	23%	Not detected*			Not detected*		Female
m.3243A>G	No pregnancies or births	23%	Not detected*			Not detected*		Male
m.3243A>G	No pregnancies or births	61%	9% and 24%			Twin 1: Not tested. Twin 2: Not detected*		Male & female
m.3688G>A	Live birth of affected child (deceased)	50%	Not detected* and 5%			7%		Female
m.8993T>G	Live birth of affected child (deceased)	not detected	Not detected*			Not detected*		Female
			Not detected*			Not detected*		Male
m.8993T>C	Live birth of affected child	37%	Not detected*			Not detected*		Female

* Not detected by pyrosequencing: Sensitivity of assay is >3%
