# Supplementary material for: Mitochondrial Donation and PGT to Reduce Risk of Mitochondrial DNA Disease
Source: N Engl J Med. Author manuscript; Available in PMC 2025 Jul 31. (PMC7617940; doi:10.1056/NEJMoa2415539)
Supplement: Supplement [file EMS206183-supplement-Supplement.pdf]

Supplementary Appendix

Mitochondrial Donation and PGT to Reduce Risk of Mitochondrial DNA Disease

Authors: Louise A. Hyslop Ph.D, Emma L. Blakely Ph.D., Magomet Aushev Ph.D., Jordan Marley Ph.D., Yuko Takeda Ph.D., Angela Pyle Ph.D., Eilis Moody M.Sc., Catherine Feeney M.Sc., Jan Dutton RGN, Carol Shaw RGN, Sarah J. Smith B.Sc., Kate Craig Ph.D., Charlotte L. Alston Ph.D., Lisa Lister Ph.D., Karina Endacott B.Sc., Samantha Byerley B.Sc., Helen McDermott B.Sc., Kathryn Wilson M.Sc., Lynne Botham B.Sc., Beth Matthew B.Sc., Nilendran Prathalingham* Ph.D, Matthew Prior Ph.D, Alison Murdoch M.D., Douglass M. Turnbull Ph.D., Gavin Hudson Ph.D., Meenakshi Choudhary Ph.D., Robert W. Taylor D.Sc, Rekha N. Pillai Ph.D., Jane A. Stewart M.D., Robert McFarland Ph.D., Mary Herbert Ph.D.

Corresponding author details: mary.herbert@newcastle.ac.uk

Table of contents

Supplementary methods 2
Figure S1. mtDNA variants and variant loads. 10
Figure S2. Patient age and egg number 11
Figure S3. Oocyte retrieval outcomes of PNT patient, PGT patient and donor eggs 12
Figure S4. Effect of vitrification and mtDNA variants on fertilization of PNT patient eggs. 13
Figure S5. PNT scoring system and number of intact zygotes for each PNT procedure. 14
Figure S6. PNT procedure outcome and PGT embryo development 15
Figure S7. Embryo transfers and clinical outcomes according to mtDNA variant type. 16
Figure S8. Pregnancy outcomes after PNT and PGT. 17
Figure S9. Heteroplasmy for maternal mtDNA in arrested PNT embryos. 18
Figure S10. Heteroplasmy for maternal mtDNA in enucleated eggs for PNT patients with heteroplasmic variants. 19
Table S1: Disease symptoms for each mtDNA pathogenic variant 20
Table S2: Representativeness of Study Participants 21
Table S3: Adverse events for pronuclear transfer (Section 33 HFEA Code of Practice) 22
Table S4: Clinical pregnancy outcome for PNT and PGT patients 23
Table S5: Primer sequences 24
References 25

Supplementary methods

Study design and oversight

The first and last authors (LH and MH) decided to publish the paper. They also designed the study, wrote the paper and vouch for the data and analysis. LH, EB, MA, JM, YT, EM, CF, JD, CS, GH, MC, RT, RP, JS, RM, MH gathered the data. LH, EB, MA, JM, YT, AP, GH, MH analyzed the data.

PNT and PGT patient data

PNT and PGT data were obtained from patients who underwent NHS funded from 2011 for PGT treatment and from 2018 for PNT treatment.

Egg donor recruitment for PNT

Egg donors for PNT were either known to the patient or recruited anonymously through the Newcastle Fertility Centre egg donation programme.

Ovarian reserve assessments

Ovarian reserve was assessed by antral follicle count (AFC) and Anti-Müllerian Hormone (AMH) levels. AMH levels were measured by Elecsys AMH assay (Roche).

Patient and egg donor screening

Patients and donors underwent screening tests including viral screen for CMV, HIV, Hepatitis B, and Hepatitis C. Donors underwent additional screening including medical evaluation and sexually transmitted infection screen for Chlamydia, Gonorrhoea and Syphilis. mtDNA sequencing for the donors was carried out in the UKAS-accredited NHS Highly Specialised Mitochondrial Diagnostic laboratory, as set out in the NHS England National Genomic Test Directory (Clinical Indication R300.1; https://www.england.nhs.uk/publication/national-genomic-test-directories/). . None of the women who donated eggs for PNT had pathogenic mtDNA variants, likely pathogenic mtDNA variants or mtDNA variants of uncertain significance. All donors were counselled in

advance of genetic testing with regard to these possible outcomes and follow up counselling was also offered. Next generation sequencing (NGS) of mtDNA using Ion Torrent technology was performed in two samples (blood and urine) to test for pathogenic mtDNA variants. Sequences were aligned to the revised Cambridge reference sequence for human mtDNA (GenBank Accession number: NC_012920.1). Data analysis was performed in Torrent Suite v5.18.1 using Variant Caller v5.18.0.0 and Coverage Analysis v5.18.0.2. 100% of the mitochondrial genome was covered at a minimum read depth of 200x. Analytical sensitivity for single nucleotide variants present at ≥5% heteroplasmy, is ≥95% (95% confidence interval). Variant classification is according to ACMG/AMP[1] and the ACGS Best Practice Guidelines for Variant Classification in Rare Disease 2024 v1.2 (www.acgs.uk.com).

Ovarian stimulation and oocyte retrieval

The ovarian stimulation regimes were tailored to the ovarian reserve markers. For antagonist cycles, highly purified hMG 150-300IU/day (11 to 12 days) and GnRH antagonist 250 mcg/day from day 5 of stimulation were self-administered. For agonist cycles, suppression of the hypothalamus/pituitary/ovarian feedback axis was achieved using GnRH agonist 500µg/day. The ovarian stimulation regime consisted of highly purified hMG 150-300IU/day for 10 to 14 days. The maturation trigger administered subcutaneously 36 hours prior to the oocyte retrieval was either hCG (250µg) or GnRH agonist (500µg or 1mg). Transvaginal ultrasound guided oocyte retrieval was performed on each occasion under sedation (100µg fentanyl IV; 3-5 mg midazolam IV). Patient oocytes containing a first polar body were deemed to be at the metaphase II stage. Metaphase II-arrested eggs were either vitrified on the day of retrieval for most PNT cases or used fresh for PGT and a small number of PNT cases. Donor eggs were used fresh on the day of the oocyte retrieval.

Oocyte vitrification and warming

Patient metaphase II-arrested eggs were vitrified and stored. Cumulus cells were removed using hyaluronidase (HYASE; Vitrolife). Rapidvit and Rapidwarm oocyte kits (Vitrolife, Sweden) were used for vitrification and warming respectively. The vitrified eggs were stored using the Rapid-I vitrification system (Vitrolife). Eggs were allowed to recover for 2-3 hours before fertilisation by intracytoplasmic sperm injection (ICSI).

Sperm freezing and ICSI

Sperm parameters were assessed according to the WHO criteria (2010). SpermFreeze (FertiPro) was used for freezing of the unprocessed semen sample. Freshly produced samples for PGT treatment and thawed semen samples for PNT treatment were centrifuged with a 2 layer Puresperm density gradient at 250 x g for 20 minutes. The pellet was washed in G-IVF medium (Vitrolife) or SpermRinse (Vitrolife) by centrifugation at 250 x g for 5 minutes and re-suspended in G-IVF or SpermRinse in preparation for ICSI.

ICSI was performed using patient partner or donor sperm. Donor eggs were fertilized with sperm from the same source as patient eggs, except in 3 cases in which the use of donor sperm was mandated because the egg donor was genetically related to the male partner.

Pronuclear transfer

Patient and donor eggs were fertilised by intracytoplasmic sperm injection (ICSI) using patient-partner or donor frozen/thawed sperm. The patient and donors eggs were monitored for signs of fertilisation from 8 hours after ICSI. PNT was conducted between normally fertilized eggs from 8-13 hours after ICSI in an environmentally controlled isolator. Patient and donor zygote pairs were placed in separate droplets of enucleation medium G-TL (Vitrolife) with Nocodazole (31.3µM) and Latrunculin A (0.625 µM), (Calbiochem). Biopsy pipettes were inserted via a laser (Saturn Active, Research Instruments)-induced hole in the zona pellucida. To minimize carryover the pronuclei were removed separately. Karyoplasts containing a single pronucleus surrounded by a small amount of cytoplasm were aspirated from the donor and patient zygotes.

After removal of the first pronucleus the procedure was repeated for the second pronucleus. The volume of cytoplasm contained in the karyoplasts was scored using the scoring scheme (Fig S4A). Each pair of patient karyoplasts were briefly exposed to a 1:10 diluted suspension of the fusogen HVJ-E (Cosmo Bio) and were gently expelled into the perivitelline space of the enucleated donor zygote. The extent of cytoplasmic leakage from the donor egg was scored using the scoring scheme (Fig S4B). The duration of exposure to enucleation medium ranged from 16-36 minutes. Karyoplast/cytoplast couplets were washed in G-TL medium (Vitrolife) and cultured in G-TL medium for 5-6 days. All procedures were conducted in environmentally controlled enclosed isolators (Walker Ltd) at 6% CO2 and the integrated RI witness heated stages at 37°C.

Preimplantation genetic testing

PGT was not performed on PNT embryos according to regulations. PGT patient eggs were fertilized by ICSI using patient-partner sperm and cultured in G-TL medium. The eggs were checked for signs of fertilization from 16 hours after ICSI and classified according to fertilisation status; normal (2PN), abnormal (1, or more than 2), unfertilised or degenerate. The normally fertilized eggs were transferred to individual drops of G-TL and cultured to day 3 after egg retrieval. Prior to biopsy individual embryos were transferred to G-PGD medium supplemented with 5% HSA (Vitrolife). The biopsy pipettes were inserted via a laser-induced hole in the zona pellucida. A single blastomere was aspirated from each embryo and released into the drop of medium containing the embryo. Following biopsy the embryos were washed in G-TL medium and cultured in individual drops of G-TL to day 5. The blastomeres were transferred into 0.5ml Eppendorf tubes and immediately frozen.

Embryo culture and grading

Embryos were assessed by light microscopy on Days 1, 3, 5 and 6. On day 1 the embryos were assessed for survival following PNT. On day 3, 5 and 6 the embryos were graded for development and quality according to UK NEQAS embryo grading scheme.

Endometrial preparation for embryo transfer

GnRH agonist (500μg/day) was administered to downregulate the pituitary/ovarian axis and synchronise the donor-patient cycle for fresh embryo transfer. Estradiol (2mg tds or qds) was given for preparation of the endometrium for embryo transfer. Transvaginal ultrasound scanning was used to confirm the endometrial thickness prior to commencing progesterone.

Embryo transfer

Progesterone (pessaries, 400mg bd alone or in addition to injection, 25mg od) was administered for luteal support from the day the eggs were warmed for PNT. Embryo transfer took place on day 5 of embryo development using a Surepro or SureView catheter (Cooper Surgical).

Embryo vitrification and warming

Blastocysts classified as top, good or fair quality according to the UK NEQAS embryo grading scheme were vitrified on days 5 and 6. Rapidvit and Rapidwarm Blast kits (Vitrolife, Sweden) were used for vitrification and warming respectively. The vitrified blastocysts were individually stored using the Rapid-I vitrification system (Vitrolife). Blastocysts were allowed to recover for up to 2 hours after warming and those which showed signs of re-expansion were transferred.

Pregnancy test and scanning

Nine days after embryo transfer either the serum hCG concentrations were measured by Elecsys Free βhCG assay (Roche) or the patient used a home pregnancy test. Ultrasound scan was used to confirm the clinical

pregnancy from 6 weeks. Those who were on estradiol and progesterone supplements continued these medications until 12 weeks gestation.

Measurement of mtDNA heteroplasmy in arrested PNT embryos

Individually frozen human embryo samples were lysed using 0.5% Tween-20, 50 mM Tris-HCl, 200 µg/ml proteinase K at 55ºC for 16 hours followed by 95ºC for 10 minutes. For the sequencing of the control region, m.3260A>G, m.3460G>A, m.11778G>A and m.4300A>G a fragment was PCR amplified using PrimeSTAR GXL DNA polymerase (Takara Bio) and primer set in Supplementary Table 2. Primer positions are in reference to the revised Cambridge reference sequence (NC_012920.1). Amplified products were assessed by gel electrophoresis, against DNA-positive and DNA-negative controls. For targeted sequencing to determine heteroplasmy, each amplicon was individually purified using Agencourt AMPure XP beads (Beckman-Coulter) and quantified using a Qubit 2.0 fluorimeter (Life Technologies). Amplicons were pooled in equimolar concentrations then libraries were prepared using Nextera XT DNA Library Prep Kit (Illumina). Libraries were sequenced using the MiSeq Reagent Kit V3 600 cycles and the Illumina MiSeq v3.0 sequencing platform (Illumina) in paired-end, 250 bp reads.

Post-run FASTQ files were analysed using an in-house-developed bioinformatic pipeline[2,3,4,5,6]. Briefly, sequence reads were quality checked using FastQC (v. 011.8), mapped against genome version GRCh38 (including NC_012920.1), using BWA (invoking –mem, v. 0.7.17)[7] sorted and indexed using Samtools (v.1.12)[8]. All duplicated reads from the resulting bam files were marked using Picard (v.2.2.4). mtDNA variants (mtSNVs) were called using bcftools (v1.10.2) and Mutserve (v.2.0.0) accordingly[9, 10]. mtSNVs with base quality (--baseQ) over 30 and minimum heteroplasmy level (--level) 0.01 were called. Low-quality variants, present in low-complexity regions[11] were not included in comparative analysis (e.g., 66–71 bp, 300–316 bp, 513–525 bp, 3106–3107 bp, 12,418–21,425 bp, 16,181–16,194 bp). Heteroplasmies were defined as the proportion of mtDNA variant allele depth relative to reference allele depth (or heteroplasmic fraction), HF. Homoplasmic variation was defined as HF>0.99 (<99%). Heteroplasmic variation was defined as HF>0.01/<0.99 (or >1% and

<98%). Coverage thresholds (compared to rCRS or GenBank ID NC_012920.1) were set at >99% at a minimal depth of 1000×.

Measurement of mtDNA heteroplasmy in blastomeres for PGT and infant peripheral blood via pyrosquencing

Pyrosequencing is the standard diagnostic test for measurement of mtDNA heteroplasmy in blastomeres for PGT and infant peripheral blood. Individually frozen blastomere samples were lysed using 0.5% Tween-20, 50 mM Tris-HCl, 200 µg/ml proteinase K at 55ºC for 2 hours followed by 95ºC for 10 minutes. DNA extraction from blood was performed according to standard protocols. Quantitative pyrosequencing (Pyromark Q24, Qiagen) using variant-specific primers was employed to determine variant heteroplasmy (test sensitivity >3% heteroplasmy; test specificity ± 2%)[12]. Data analysis was performed using PyroMark Q24 software (v2.0.8).

Measurement of mtDNA heteroplasmy in infant peripheral blood via next generation sequencing

NGS was performed in a number of cases for example to assess the efficacy of the PNT technique when the pyrosequencing diagnostic result was undetectable. The appropriate region of the mitochondrial genome was amplified by long-range PCR and Next Generation sequencing was performed using Ion Torrent technology. Sequences were aligned to the revised Cambridge reference sequence for human mtDNA (Nature Genet. 1999;23:147, GenBank Accession number: NC_012920.1). Read depth data analysis for the familial variant was performed using a custom in-house Python script.

Control data sets

ICSI control data (presented in Fig. S2) were obtained from patients who underwent ICSI treatment for male factor infertility. Ovarian reserve assessment, screening, ovarian stimulation, oocyte retrieval, sperm preparation, ICSI, embryo culture and embryo transfer procedures were performed as outlined above.

Adverse events

Adverse events for mitochondrial donation are defined by the HFEA: HFEA Code of Practice, Section 33, Edition 9.4. October 2023 (Table S3).

Statistical analysis

Data were analyzed using Chi-square test, Fisher's exact test, unpaired t test and Tukey's multiple comparison test in GraphPad Prism v9.0.1, as indicated in the figure legends. Means presented are ± the standard deviation (sd).

Figure S1. mtDNA variants and variant loads.

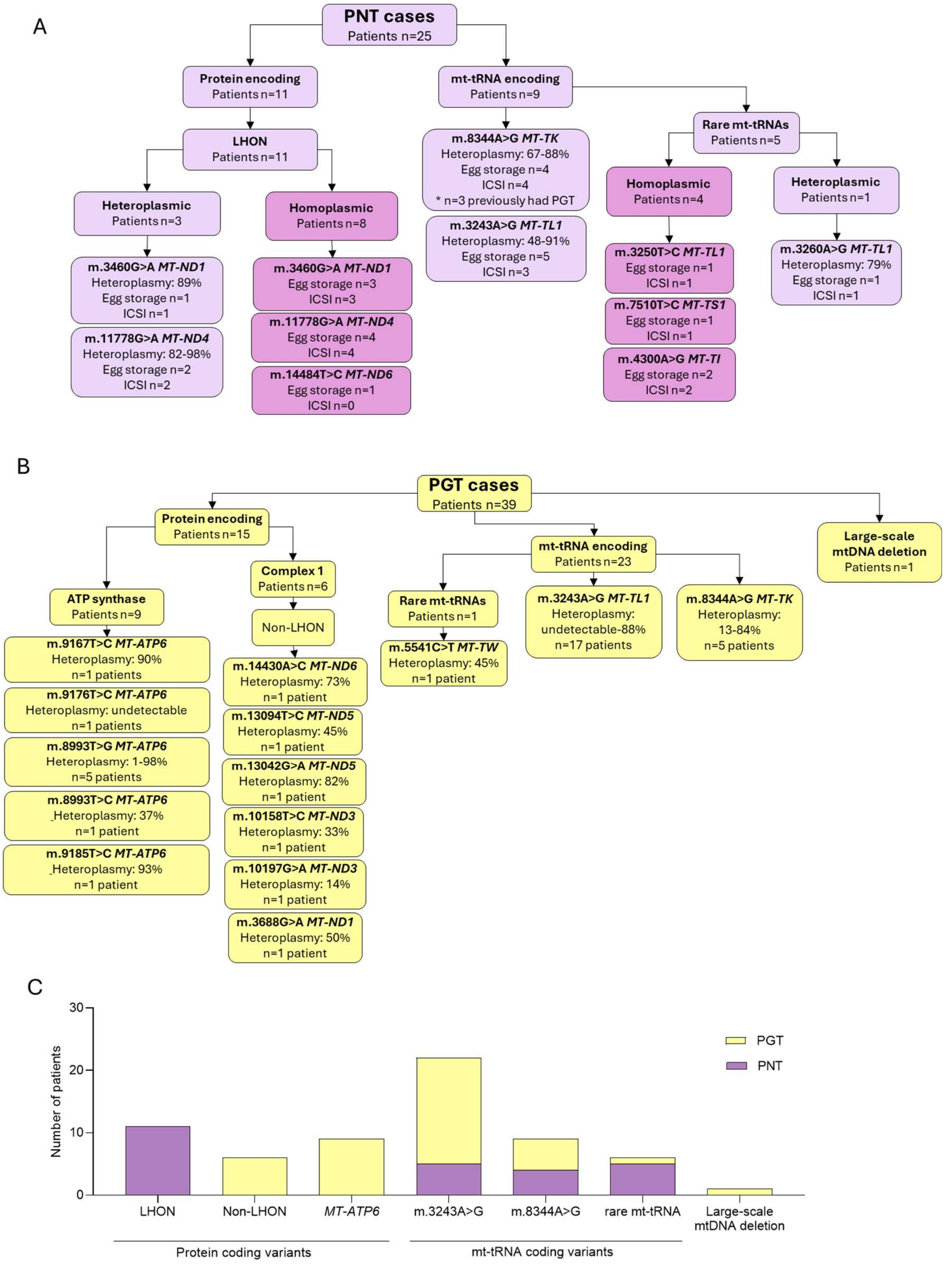

Figure S1. mtDNA variants and variant loads. (A) Illustration of mtDNA variants and variant loads of PNT patients. Heteroplasmic mtDNA variants in light purple boxes and homoplasmic mtDNA variants in dark purple boxes. N numbers are the number of patients. (B) Illustration of mtDNA variants and variant loads of PGT patients. N numbers are the number of patients. (C) Number of patients with each type of variant.

Figure S2. Patient age and egg number

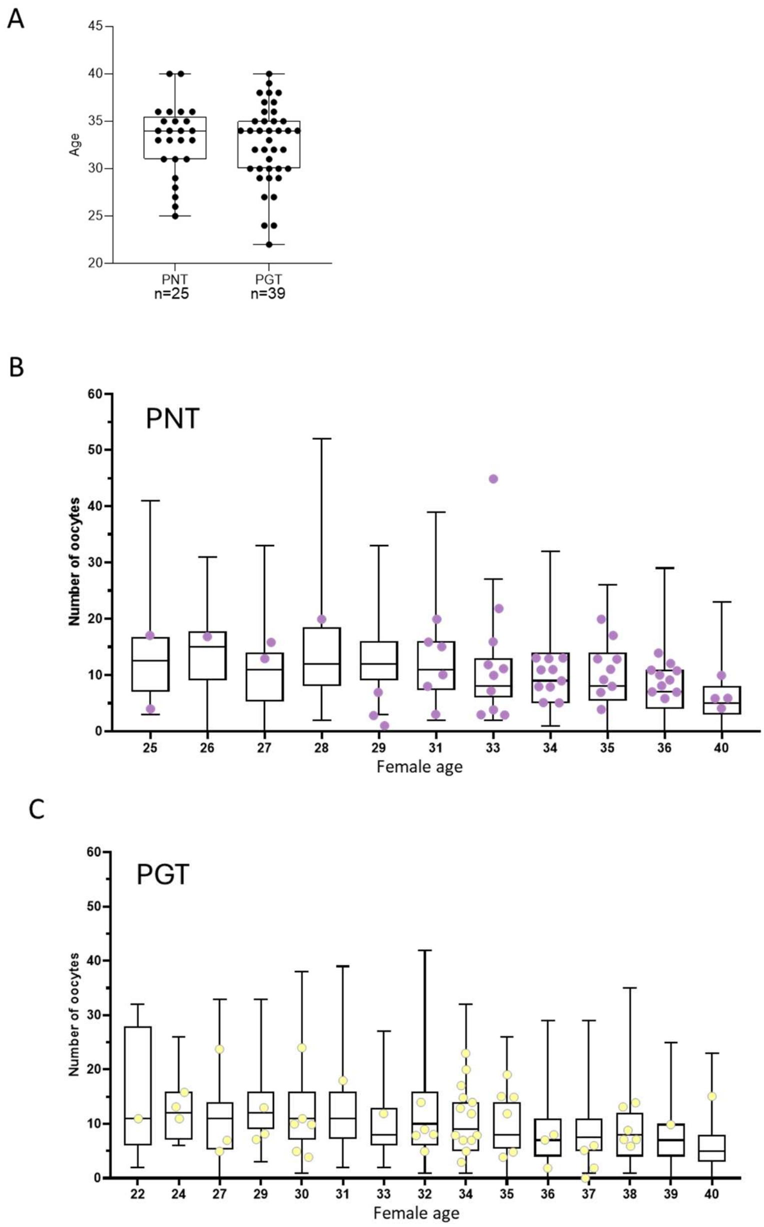

Figure S2. Patient age and egg number. (A) Comparison of PNT and PGT patient ages. (B) Box and whisker plots showing that the number of oocytes obtained per oocyte retrieval for PNT patients was comparable with age-matched control patients who underwent ICSI for male infertility. Box and whisker show age-matched control data and purple dots shows all PNT patient data. (C) Box and whisker plots showing that the number of oocytes obtained per oocyte retrieval for PNT patients was comparable with age-matched control patients who underwent ICSI for male infertility. Box and whisker show age-matched control data and yellow dots shows all PGT patient data.

Figure S3. Oocyte retrieval outcomes of PNT patient, PGT patient and donor eggs.

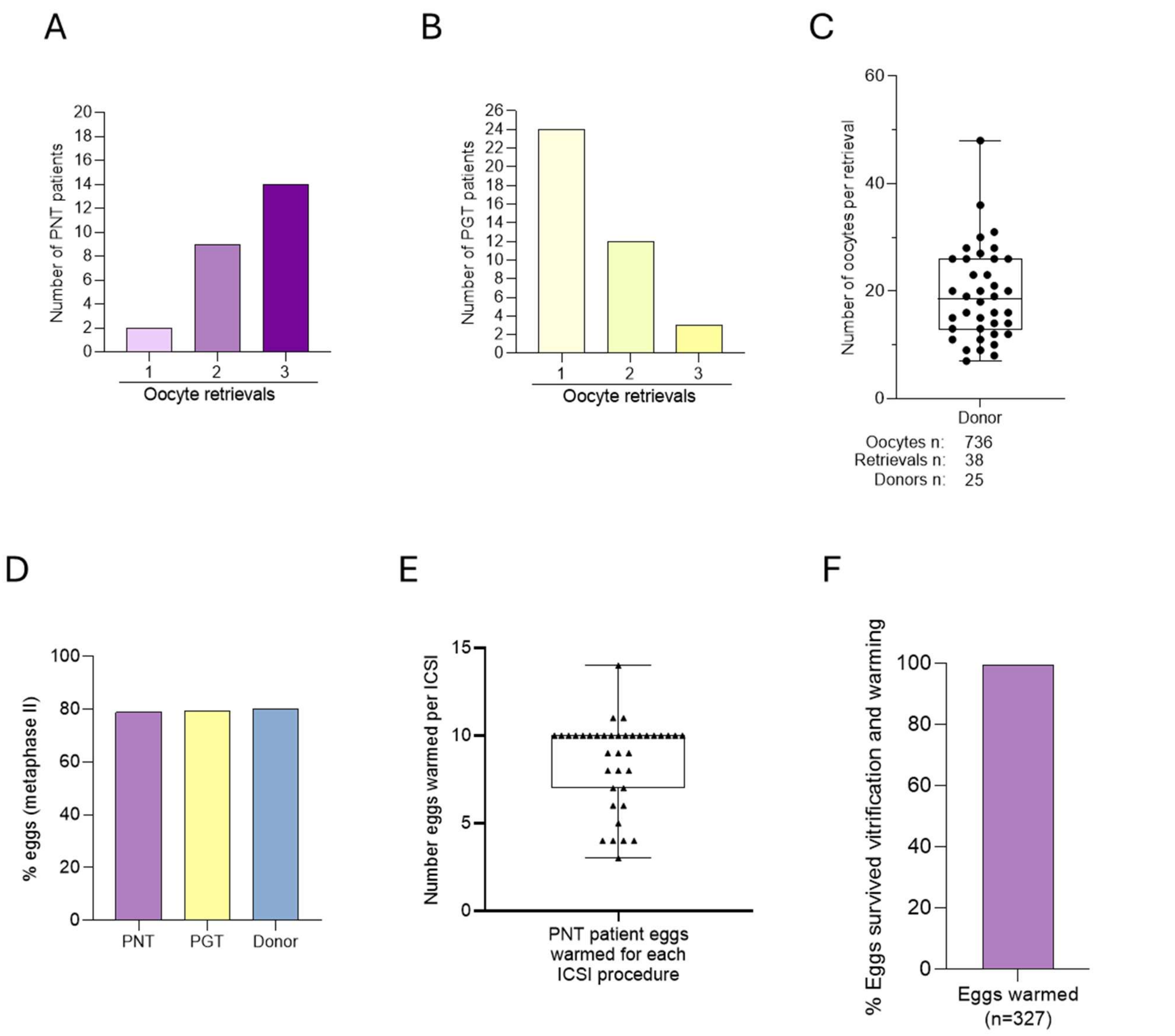

Figure S3. Oocyte retrieval outcomes of PNT patient, PGT patient and donor eggs. (A) Number of PNT patients undergoing 1, 2 or 3 rounds of egg retrieval. (B) Number of PGT patients undergoing 1, 2 or 3 rounds of egg retrieval. (C) Number of oocytes per egg retrieval for egg donors. (D) Proportion of mature eggs obtained from PNT patients, PGT patients and egg donors. (E) Number of patient eggs warmed per ICSI for PNT. (F) Proportion of eggs that survived vitrification and warming.

Figure S4. Effect of vitrification and mtDNA variants on fertilization of PNT patient eggs.

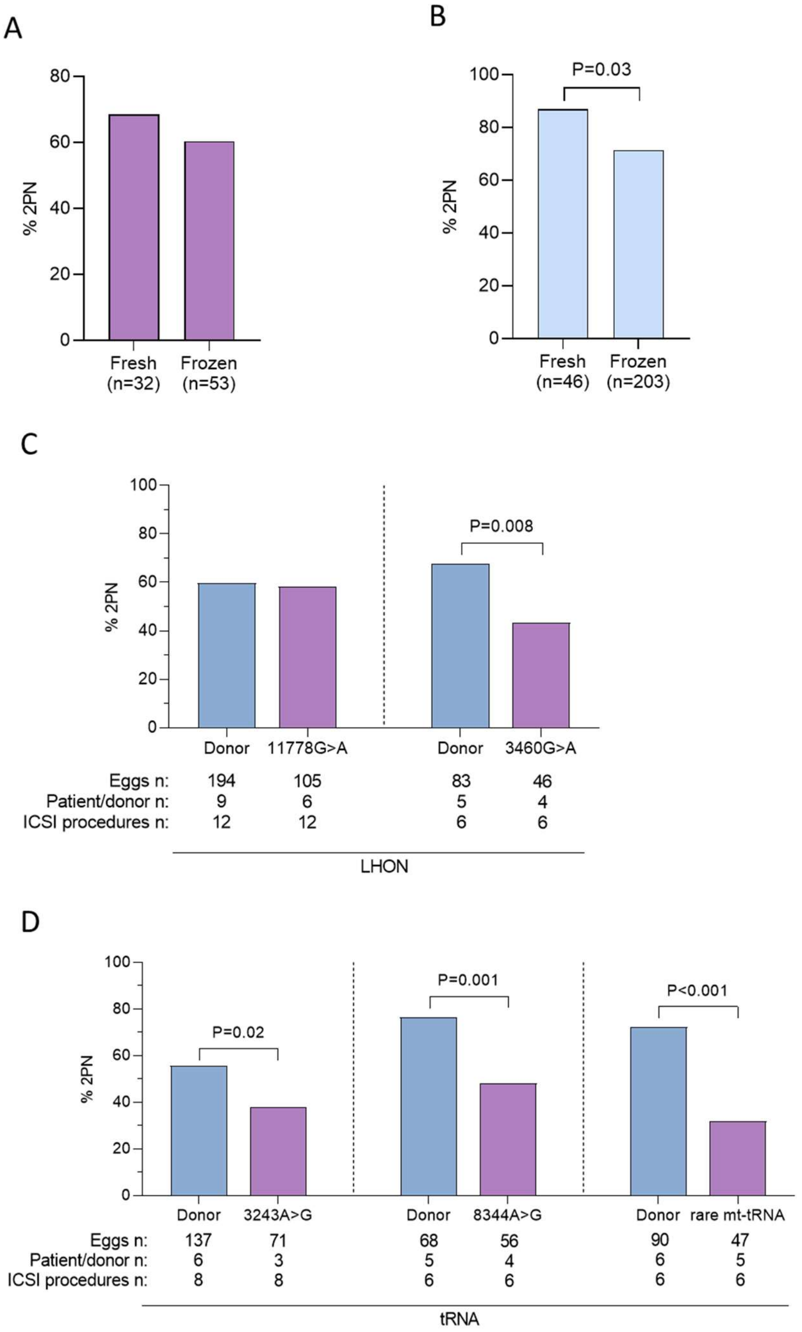

Figure S4. Effect of vitrification and mtDNA variants on fertilization of PNT patient eggs. (A) Graph showing no difference in the proportions of normally fertilized fresh and frozen (vitrified) eggs from PNT patients (n=4). (B) Graph showing reduced fertilization of vitrified eggs in a larger series of eggs donated for research (P=0.03; Chi-Square). (C) Graph showing the proportions of eggs carrying LHON variants that underwent normal fertilization. Eggs with the *MT-ND1* m.3460G>A variant had reduced fertilization compared with donor eggs (P=0.008; Chi-Square). (D) Graph showing the proportions of eggs carrying variants in mt-tRNA-coding sequences that underwent normal fertilization. All had reduced proportions of normally fertilized eggs compared with donor eggs (Chi-Square). The most profound reduction was observed in cases with rare mt-tRNA variants (P<0.001; Chi-Square).

Figure S5. PNT scoring system and number of intact zygotes for each PNT procedure.

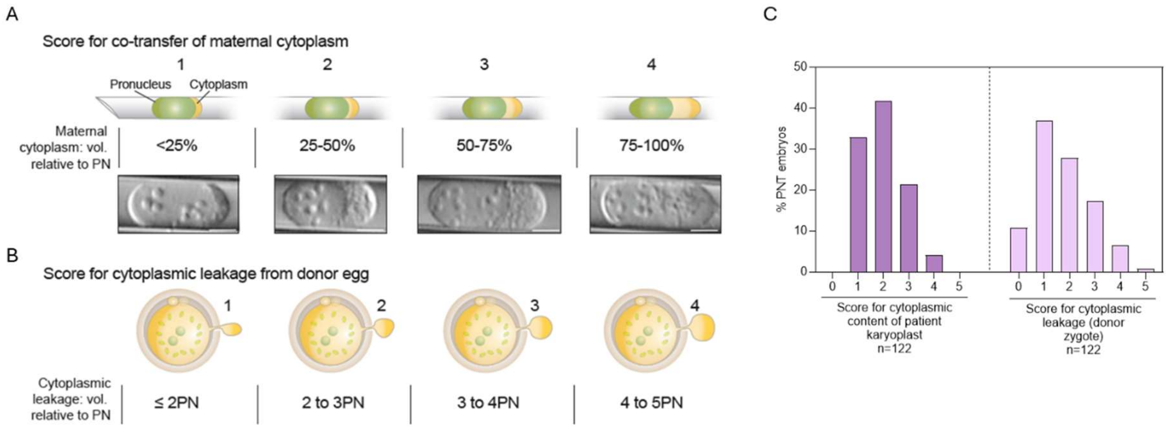

Figure S5. PNT scoring system and number of intact zygotes for each PNT procedure. (A) Schematic illustrating the scoring system used to estimate the volume of cytoplasm relative to the volume of the pronucleus in each patient karyoplast. (B) Schematic illustrating the scoring system used to estimate the volume of cytoplasmic leakage from the donor egg relative to the volume of the pronucleus. (C) The frequency and scores 1-5 for cytoplasmic content of patient karyoplast and donor egg cytoplasmic leakage for all PNTs (n=122) performed.

Figure S6. PNT procedure outcome and PGT embryo development.

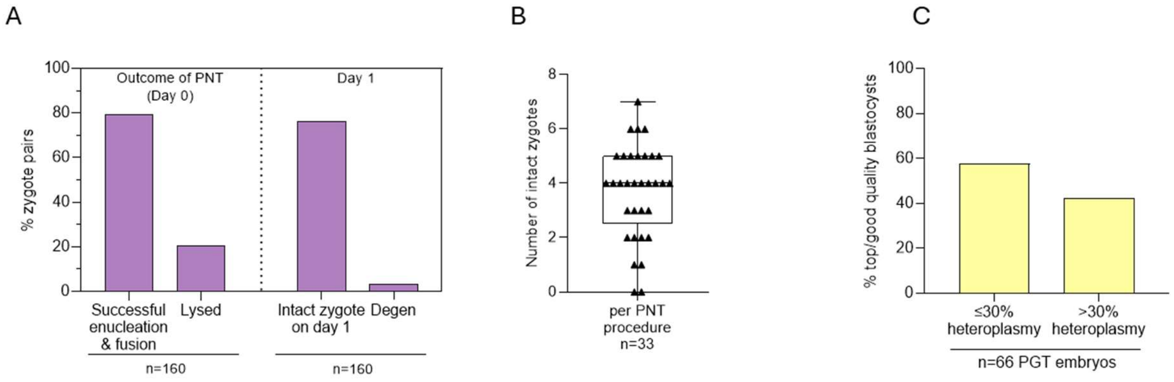

Figure S6. PNT procedure outcome and PGT embryo development. (A) Graphs showing fusion outcomes and intact zygotes on day 1 after PNT. (B) Graph showing the number of intact zygotes obtained per PNT procedure. (C) Proportions of top and good quality PGT blastocysts with ≤30% and >30% heteroplasmy for the maternal pathogenic variant.

Figure S7. Embryo transfers and clinical outcomes according to mtDNA variant type.

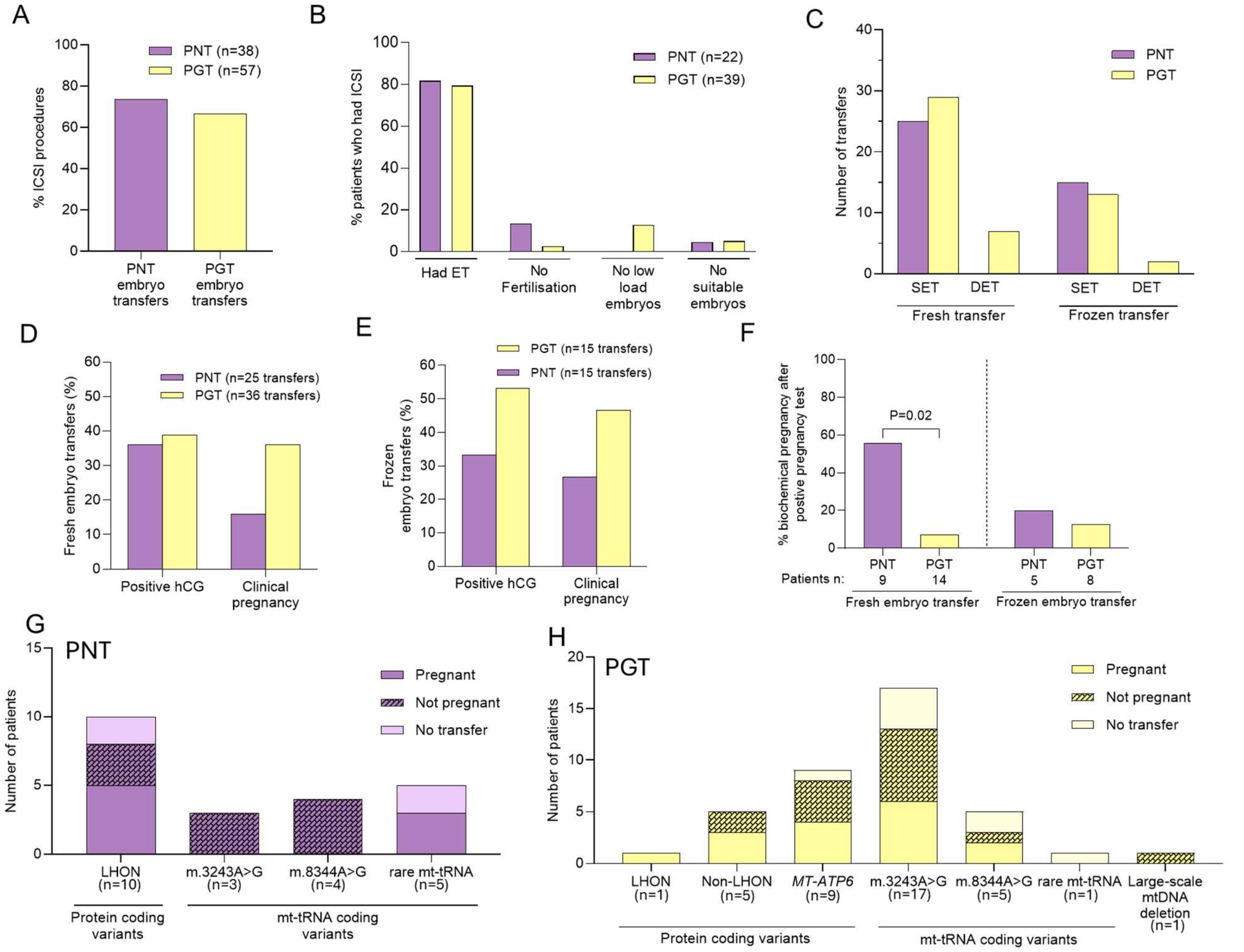

Figure S7. Embryo transfers and clinical outcomes according to mtDNA variant type. (A) Graph showing proportion of ICSI procedures resulting in embryo transfers after PNT and PGT. (B) Graph showing reasons why patients did not have an embryo transfer after PNT an PGT. (C) Graph showing the number of single and double embryo transfers for PNT and PGT patients. (D) Graph showing the proportions of fresh embryo transfers that resulted in a positive pregnancy test and clinical pregnancy after PNT and PGT. (E) Graph showing the proportions of frozen embryo transfers that resulted in a positive pregnancy test and clinical pregnancy after PNT and PGT. (F) Graph showing proportion of biochemical pregnancies after a positive pregnancy test for fresh and frozen embryo transfers after PNT and PGT (Fishers exact). The incidence of pregnancy loss after a positive pregnancy test was increased after a fresh PNT transfer compared with fresh PGT transfer (P=0.02; Fisher's exact). (G) Graph showing treatment outcome per PNT patient according to mtDNA variant type. (H) Graph showing treatment outcome per PGT patient according to mtDNA variant type.

Figure S8. Pregnancy outcomes after PNT and PGT.

A

PNT				
**Variant**	**% heteroplasmy maternal urine**	**Sex**	**Gestation (weeks)**	**Birth weight centile**
m.4300A>G	Homoplasmic	Female	38	25th-50th centile
m.4300A>G	Homoplasmic	Male	38	25th-50th centile
m.3260A>G	79%	Male	38	25th-50th centile
m.3460G>A	89%	Female	38	25th-50th centile
m.11778G>A	82%	Female	42	50th-75th centile
m.11778G>A	Homoplasmic	Identical twin males	36	25th-50th centile
				9th-25th centile
m.11778G>A	98%	Female	36	75th-91st centile
**PGT**				
**Variant**	**% heteroplasmy maternal urine**	**Sex**	**Gestation (weeks)**	**Birth weight centile**
m.3243A>G	Not detected	Female	39	9th-25th centile
m.3243A>G	6%	Male	41	25th-50th centile
		Female	40	Not known
m.3243A>G	23%	Female	40	50th-75th centile
m.3243A>G	23%	Male	35	50th-75th centile
m.3243A>G	61%	Male & female (twins)	36	Not known
m.8344A>G	13%	Male	38	Not known
m.8344A>G	70%	Male	39	50th-75th centile
m.10158T>C	33%	Male	40	9th-25th centile
		Female	39	25th-50th centile
m.13094T>C	45%	Male	38	50th-75th centile
m.3688G>A	50%	Female	40	50th-75th centile
m.8993T>G	Not detected	Female	40	75th-91st centile
		Male	36	75th-91st centile
m.8993T>C	37%	Female	39	25th-50th centile
m.8993T>G	64%	Male	39	Not known
m.9167T>C	90%	Female	36	9th-25th centile

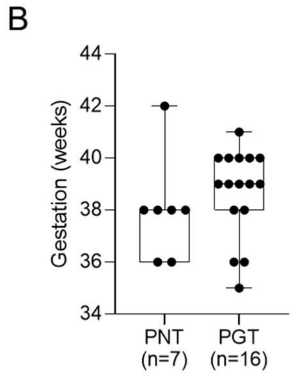

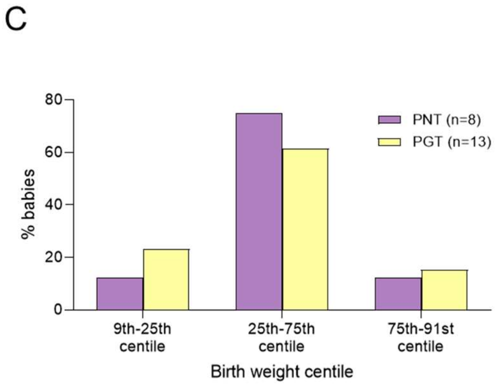

Figure S8. Pregnancy outcomes after PNT and PGT. (A) Table shows gestation and birth weight of babies born following PNT and PGT. (B) Graph showing no significant difference in gestation in weeks for PNT and PGT singleton babies born (P=0.68; unpaired t test). (C) Graph showing no significant difference in birth weight centiles between PNT and PGT babies (Fisher's Exact). The UK-WHO growth charts for girls and boys were used to determine the birth weight centiles.

Figure S9. Heteroplasmy for maternal mtDNA in arrested PNT embryos.

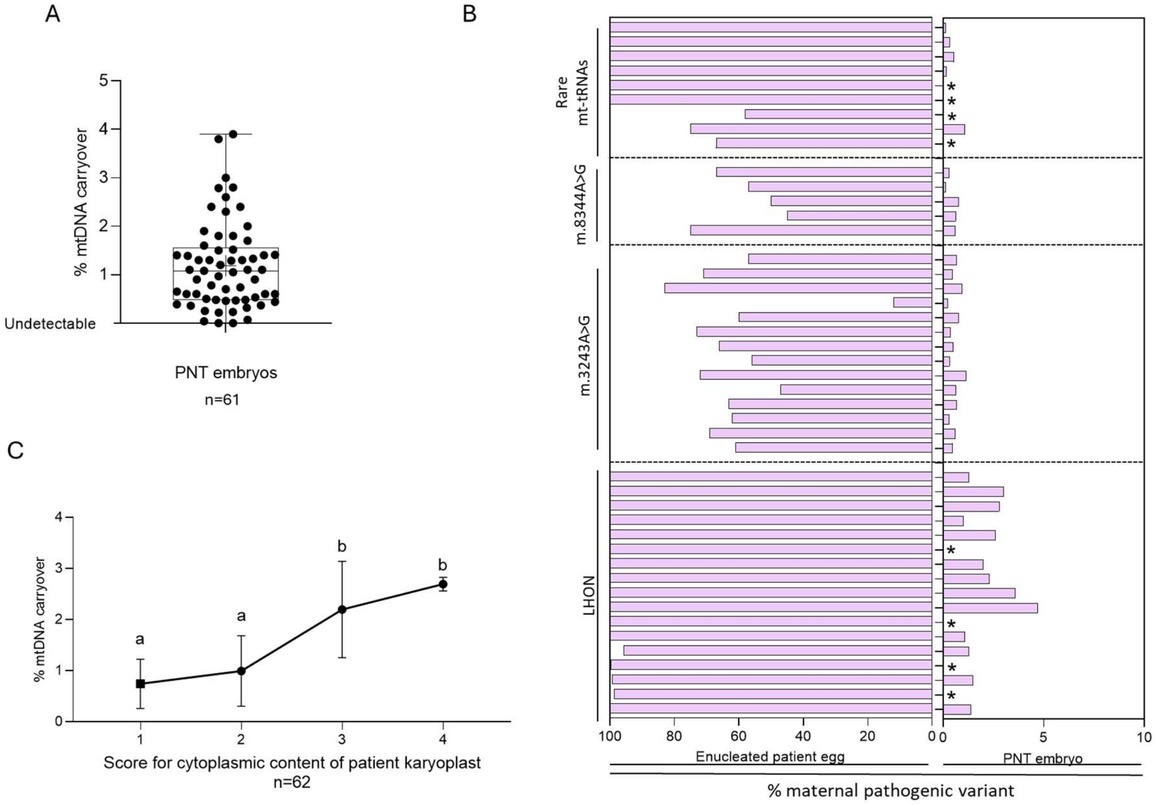

Figure S9. Heteroplasmy for maternal mtDNA in arrested PNT embryos. (A) Heteroplasmy due to carryover of maternal mtDNA in arrested PNT embryos (n= 62). (B) Graph showing heteroplasmy measured by NGS in the enucleated patient egg versus the resultant arrested PNT embryo. * indicates the pathogenic variant was not detected by NGS.(C) Levels of heteroplasmy according to the estimated volume of maternal cytoplasm contained in the patient karyoplast. Lowercase letters indicate significant differences (P<0.01; Tukeys multiple comparison test).

Figure S10. Heteroplasmy for maternal mtDNA in enucleated eggs for PNT patients with heteroplasmic variants.

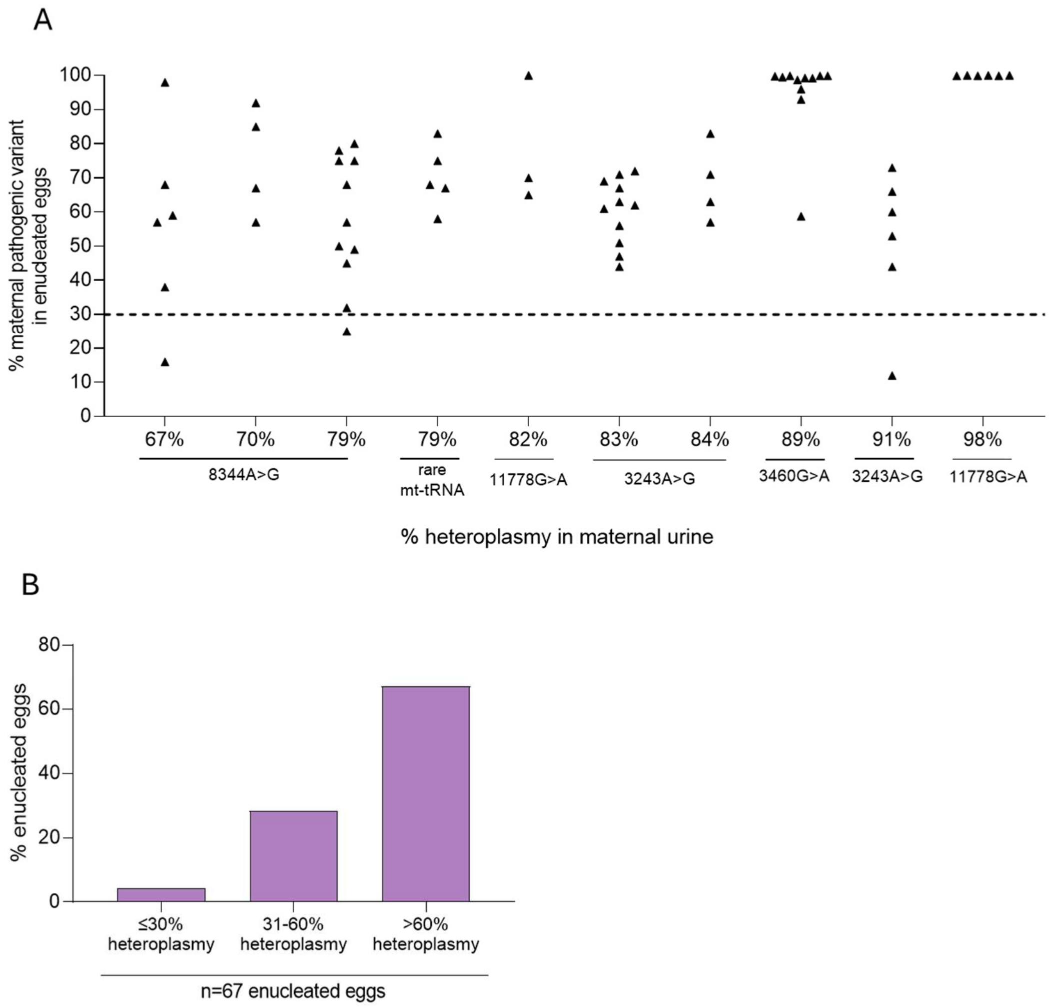

Figure S10. Heteroplasmy for maternal mtDNA in enucleated eggs for PNT patients with heteroplasmic variants. (A) Graph showing heteroplasmy measured by NGS in enucleated eggs from 10 of 11 PNT patients with heteroplasmic variants. The dotted line shows the 30% threshold. (B) Graph showing the proportion of enucleated eggs with ≤30%, 31-60% and >60% heteroplasmy.

Table S1: Disease symptoms for each mtDNA pathogenic variant

mtDNA pathogenic variant #			Number of patients	Disease symptoms
Genes encoding mt-tRNAs	m.3243A>G (*MT-TL1*)		21	Multisystem disease including;<br>• Cardiomyopathy<br>• Myopathy<br>• Ataxia<br>• Diabetes<br>• MELAS: Mitochondrial encephalomyopathy with lactic acidosis and stroke-like episodes
	m.8344A>G (*MT-TK*)		6	Multisystem disease including;<br>• Myoclonus<br>• Seizures<br>• Cerebellar ataxia<br>• Myopathy
	Rare mt-tRNAs	m.3250T>C (*MT-TL1*)	1	Skeletal myopathy with lifetime risk of cardiomyopathy
		m.3260A>G (*MT-TL1*)	1	Cardiomyopathy with or without skeletal myopathy
		m.4300A>G (*MT-TI*)	2	Hypertrophic cardiomyopathy
		m.5541C>T (*MT-TW*)	1	Stroke-like episodes. Seizures &/or dementia
		m.7510T>C (*MT-TS1*)	1	Hearing Loss and a range of neurological defects including myopathy and ataxia
MT-ND genes encoding complex I subunits	m.3460G>A (*MT-ND1*)		4	LHON: Leber hereditary optic neuropathy
	m.11778G>A (*MT-ND4*)		6	
	m.14484T>C (*MT-ND6*)		1	
	m.10158T>C (*MT-ND3*)		1	Stroke-like episodes
	m.3688G>A (*MT-ND1*)		1	Leigh syndrome
	m.10197G>A (*MT-ND3*)		1	
	m.13094T>C (*MT-ND5*)		1	
	m.14430A>C (*MT-ND6*)		1	
	m.13042G>A (*MT-ND5*)		1	Stroke like episodes, optic atrophy and ataxia.
Gene encoding ATP synthase	m.8993T>G (*MT-ATP6*)<br>m.8993T>C (*MT-ATP6*)		6	NARP and Leigh syndrome<br>• Childhood or early adult-onset peripheral neuropathy<br>• Ataxia<br>• Retinitis pigmentosa
	m.9167T>C (*MT-ATP6*)		1	Leigh syndrome
	m.9176T>C (*MT-ATP6*)		1	
	m.9185T>C (*MT-ATP6*)		1	
Large-scale mtDNA deletion (m.7529_14025del)			1	Multisystem disease including;<br>• Myopathy<br>• External ophthalmoplegia<br>• Cardiomyopathy<br>• Ataxia

mtDNA variant positions are in reference to the revised Cambridge reference sequence (NC_012920.1)

Table S2: Representativeness of Study Participants

Category	
Disease, problem, or condition under investigation	Mitochondrial DNA disease an umbrella term that encompasses a group of disorders caused by mutations in mitochondrial DNA (mtDNA), also known as Primary Mitochondrial Disease.
Special considerations related to	Mitochondrial DNA disease is transmitted exclusively through the female germline; females can pass the disease on to their children but males cannot.
Sex and gender	Mitochondrial DNA disease affects both males and females although with some pathogenic variants the disease more frequently manifests in men. For example Leber's Hereditary Optic Neuropathy, which shows a male:female bias (5:1).
Age	mtDNA disease is a genetic condition that can present at any age. The prevalence increases with age.
Race or ethnic group	mtDNA disease affects all races and ethnic groups.
Geography	mtDNA variation is strongly influenced by geography, with distinct haplogroups and disease-associated mutations distributed according to ancient migrations, and population history. This structuring affects the prevalence and manifestation of mtDNA disease in different regions of the world.<br><br>For example, in the North East of England the prevalence of LHON is estimated at 3.7 per 100,000, compared with 1.85 per 100,000 in Denmark, 0.3 per 100,000 in New Zealand, 2 in 100,000 in the US and <0.1 100,000 in Africa.
Other considerations	PGT and mitochondrial donation are available to women at risk of transmitting mitochondrial DNA disease to their children. Mitochondrial donation is available only to those for whom PGT is considered unsuitable. This includes women with homoplasmic pathogenic variants or high levels of heteroplasmy. In the UK mitochondrial donation treatment requires case-specific approvals from Human Fertilisation and Embryology Authority Statutory Approvals Committee.
Overall representativeness of this treatment	The clinical service is available free to all women in the UK with pathogenic mtDNA variants. The patients described in this study were all female and living in the UK. Those progressing to PGT, or mitochondrial donation had to satisfy specific criteria related to female age, ovarian reserve and health consequences of treatment, pregnancy and childbirth.<br><br>The ethnicity of women included this series of patients in our was 91.7% White and 8.3% Asian. For reference, based on the 2021 census in England and Wales: 82% were White, 9% Asian, 4% Black, 3% Mixed and 2% Others. For our study information on race and ethnicity is available through the medical records.

Table S3: Adverse events for pronuclear transfer (Section 33 HFEA Code of Practice)

Event	Pronuclear transfer patients n=25	Egg donors n=25	PGT patients n=39
Severe / critical case of ovarian hyperstimulation syndrome	0	0	0
Failed or no embryo development (%)	4 (16%)	NA	3 (7.7%)
Miscarriage (%)	0	NA	3 (7.7%)
Premature birth: delivery before the 37th week of pregnancy (%)	2 (8%)	NA	4 (10.3%)
Child born with a genetic abnormality	0	NA	0
Child born with a birth defect	0	NA	0
Child born with a mitochondrial disease	0	NA	0

Footnote: Adverse events relating to child health outcomes are described by McFarland et al. in an accompanying report in this issue of the Journal.

Table S4: Clinical pregnancy outcome for PNT and PGT patients

PNT				
Variant	% heteroplasmy<br>Maternal urine	Age	Reproductive history	PNT treatment outcome
m.4300A>G	Homoplasmic	33	No pregnancies or births	Live birth
m.4300A>G	Homoplasmic	36	Live birth of affected child (deceased)	Live birth
m.3260A>G	79%	28	No pregnancies or births	Live birth
m.3460G>A	89%	31	No pregnancies or births	Live birth
m.11778G>A	82%	34	No pregnancies or births	Live birth
m.11778G>A	Homoplasmic	31	No pregnancies or births	Live birth (monozygotic twins)
m.11778G>A	98%	31	Live birth	Live birth
m.11778G>A	Homoplasmic	33	No pregnancies or births	Ongoing pregnancy
**PGT**				
**Variant**	**% heteroplasmy<br>Maternal urine**	**Age**	**Reproductive history**	**PGT treatment outcome**
m.3243A>G	Not detected	40	No pregnancies or births	Live birth
m.3243A>G	6%	30	No pregnancies or births	2 live birth
m.3243A>G	23%	34	No pregnancies or births	Live birth
m.3243A>G	23%	24	No pregnancies or births	Live birth
m.3243A>G	58%	29	No pregnancies or births	Miscarriage
m.3243A>G	61%	31	No pregnancies or births	Live birth (twins)
m.8344A>G	13%	33	No pregnancies or births	Live birth
m.8344A>G	70%	32	2 live births	Live birth
m.10158T>C	33%	32	Live birth of affected child (deceased)	2 live births and a miscarriage
m.13042G>A	82%	30	No pregnancies or births	Miscarriage
m.13094T>C	45%	24	Live birth of affected child (deceased)	Live birth
m.3688G>A	50%	35	Live birth of affected child (deceased)	Live birth
m.8993T>G	not detected	34	Live birth of affected child (deceased)	2 live births
m.8993T>C	37%	37	Live birth of affected child	Live birth
m.8993T>G	64%	22	No pregnancies or births	Live birth
m.9167T>C	90%	32	Miscarriage	Live birth

Footnote: Further detailed clinical information can be found in the McFarland et al paper.

Table S5: Primer sequences

Primer sequences			
**Primers for mtDNA variant heteroplasmy analysis**			
**Fragment**	**Primer**	**Position**	**Sequence**
Control region	Forward	m.15758-15777	5’-ATCGGAGGACAACCAGTAAG-3’
	Reverse	m.1248-1268	5’-GATGGCGGTATATAGGCTGAG-3’
m.3260A>G, m.3460G>A, m.4300A>G	Forward	m.2395-2415	5’-ACCAACAAGTCATTATTACCC-3’
	Reverse	m.4627-4646	5’-ATACTTGATGGCAGCTTCTG-3’
m.11778G>A	Forward	m.9974-9994	5’- CTATTGATGAGGGTCTTACTC-3’
	Reverse	m.12196-12216	5’- GAGCTTTCTCGGTAAATAAGG-3’

References

1) Richards S, Aziz N, Bale S, et al. Standards and guidelines for the interpretation of sequence variants: a joint consensus recommendation of the American College of Medical Genetics and Genomics and the Association for Molecular Pathology. Genet Med. 2015;17(5):405-24.
2) Hudson G, Takeda Y, Herbert M. Reversion after replacement of mitochondrial DNA. Nature. 2019;574(7778):E8-E11.
3) Bury A, Pyle A, Vincent AE, Actis P, Hudson G. Nanobiopsy investigation of the subcellular mtDNA heteroplasmy in human tissues. Sci Rep. 2024;14(1):13789.
4) Coxhead J, Kurzawa-Akanbi M, Hussain R, Pyle A, Chinnery P, Hudson G. Somatic mtDNA variation is an important component of Parkinson's disease. Neurobiol Aging. 2016;38:217.e1-217.e6.
5) Floros VI, Pyle A, Dietmann S, Wei W, Tang WCW, et al. Segregation of mitochondrial DNA heteroplasmy through a developmental genetic bottleneck in human embryos. Nat Cell Biol. 2018 Feb;20(2):144-151.
6) Hipps D, Pyle A, Porter ALR, et al. Variant load of mitochondrial DNA in single human mesenchymal stem cells. Sci Rep. 2024;14(1):20989.
7) Li, H. & Durbin, R. Fast and accurate short read alignment with Burrows--Wheeler transform. Bioinformatics 25, 1754–1760 (2009).
8) Li H, Handsaker B, Wysoker A, et al. 1000 Genome Project Data Processing Subgroup, The Sequence Alignment/Map format and SAMtools, *Bioinformatics*, 2009;25(16):2078–2079
9) Heng Li, A statistical framework for SNP calling, mutation discovery, association mapping and population genetical parameter estimation from sequencing data, *Bioinformatics*, 2011;27(21):2987–2993
10) Weissensteiner, H., Forer L, Fuchsberger C et al*.* mtDNA-Server: Next-generation sequencing data analysis of human mitochondrial DNA in the cloud. *Nucleic Acids Res.* 2016*;*44:W64-69.

11) Goto, H., Dickins, B., Afgan, E. et al. Dynamics of mitochondrial heteroplasmy in three families investigated via a repeatable re-sequencing study. Genome Biol 2011:12:R59

12) Mavraki, E., Labrum, R., Sergeant, K. *et al.* Genetic testing for mitochondrial disease: the United Kingdom best practice guidelines. *Eur J Hum Genet* 2023;31:148–163
