# Supplementary material for: Mitochondrial Donation and PGT to Reduce Risk of Mitochondrial DNA Disease
Source: N Engl J Med. Author manuscript; Available in PMC 2025 Jul 31. (PMC7617940; doi:10.1056/NEJMoa2415539)
Supplement: Supplementary Video Legends [file EMS206183-supplement-Supplementary_Video_Legends.pdf]

Video Legends

Movie 1. Removal of pronuclei. The pronuclei are removed separately. The movie shows the enucleation pipette being inserted through the laser-drilled hole in the zona. The first pronucleus is aspirated in a karyoplast surrounded by a small amount of cytoplasm and a fragment of the egg membrane. The enucleation pipette is reinserted through the hole in the zona and the second pronucleus is aspirated into the pipette in a karyoplast.

Movie 2. Replacement of pronuclei. The movie shows the two karyoplasts, each containing a pronucleus being placed together under the zona of an enucleated donor egg by inserting the enucleation pipette through the laser-drilled hole in the zona.
